# Mechanism of Piezo1 regulating chondrocyte mitochondrial function and promoting fracture healing through β-catenin/LARS2 signaling pathway

**DOI:** 10.1038/s41413-025-00459-4

**Published:** 2025-09-24

**Authors:** Tao Zhang, Hongzhi Lv, Siming Jia, Lijun Wang, Weijian Liu, Kai Ding, Xiaofeng Du, Guangzhao Hou, Zhiyong Hou, Yingze Zhang, Weiguo Zou, Wei Chen, Yanbin Zhu

**Affiliations:** 1 Department of Orthopaedic Surgery, Hebei Medical University Third Hospital, Shijiazhuang, Hebei China; 2 Key Laboratory of Biomechanics of Hebei Province, Shijiazhuang, Hebei China; 3 NHC Key Laboratory of Intelligent Orthopaedic Equipment, Shijiazhuang, Hebei China; 4 Hainan Institute of Regenerative Orthopedics and Sports Medicine, Hainan Academy of Medical Sciences and School of Basic Medicine, Hainan Medical University, Haikou, Hainan China; 5 Key Laboratory of RNA Innovation, Science and Engineering, CAS Center for Excellence in Molecular Cell Science, Shanghai Institute of Biochemistry and Cell Biology, University of Chinese Academy of Sciences Chinese Academy of Sciences, Shanghai, China; 6 Department of Orthopaedics Union Hospital Tongji Medical College Huazhong University of Science and Technology, Wuhan, Hubei China; 7 Engineering Research Center of Orthopedic MinimallyInvasive Intelligent Equipment, Ministry of Education, Shijiazhuang, Hebei China

**Keywords:** Bone, Pathogenesis

## Abstract

Piezo1, a key mechanosensor in bone homeostasis, plays a crucial role in fracture healing. However, the mechanisms through which Piezo1 regulates chondrocytes and affects endochondral ossification remain poorly understood. This study aimed to investigate the regulatory mechanisms of Piezo1 in chondrocytes during endochondral ossification. Using lineage tracing, we identified chondrocyte-to-osteoblast transdifferentiation during endochondral ossification, which was impaired by chondrocyte-specific Piezo1 knockout. Piezo1 deficiency disrupted mitochondrial bioenergetics, characterized by diminished membrane potential, reduced adenosine triphosphate (ATP) synthesis, suppressed oxygen consumption rates (basal and maximal respiration), and elevated mitochondrial superoxide generation, thereby impairing endochondral ossification during fracture healing. Single-cell RNA sequencing revealed upregulated Lars2 expression in hypertrophic chondrocytes following Piezo1 knockout. Inhibition of Lars2 in chondrocytes normalized mitochondrial dynamics-related markers (MFN1, MFN2, OPA1, DRP1) and restored mitochondrial functional homeostasis. This intervention concurrently reversed Piezo1 knockout-induced suppression of osteogenic markers (Col1, ALP, OCN, OPN, RUNX2), thereby enhancing fracture repair. Protein interaction analyses confirmed direct binding between β-catenin and Lars2. Mechanistically, Piezo1 governs Lars2 expression via β-catenin signaling. Our findings demonstrate that Piezo1 activation via Yoda1 enhances mitochondrial bioenergetics and accelerates fracture repair through the β-catenin/Lars2 axis, offering novel insights and therapeutic avenues for fracture treatment.

## Introduction

As globally prevalent musculoskeletal injuries, fractures result in delayed or non-union complications in 5%–10% of hospitalized cases, imposing substantial socioeconomic burdens due to prolonged treatment and disability.^1,2^ Despite advancements in treatment, the regulatory mechanisms of fracture healing remain unclear, and there are currently no targeted drugs to promote fracture healing.^3^ Therefore, investigating the precise molecular mechanisms underlying fracture healing, reducing the risk of delayed and non-union fractures, and identifying potential intervention targets are critical scientific challenges that need to be addressed. Consequently, these efforts are of clinical importance.

During fracture healing, callus formation occurs via two primary pathways: intramembranous ossification and endochondral ossification.^4^ Endochondral ossification predominantly occurs in long bones (e.g., appendicular and axial skeletal elements) and represents a critical mechanism of fracture repair.^5^ During the initial phase of fracture repair, multipotent stem cells, particularly periosteal stem cells (PSCs) and bone marrow mesenchymal stem cells (BMSCs), migrate to the injury site and commit to chondrogenic differentiation.^6–9^ Conventional models of endochondral ossification posits that chondrocytes undergo hypertrophic differentiation, and hypertrophic chondrocytes (HTCs) subsequently undergo apoptosis and removal by osteoclasts during fracture healing.^9^ Emerging evidence has suggested that chondrocytes can also transdifferentiate into osteoblasts during fracture healing.^10^ Piezo1, a mechanosensitive channel protein expressed in various cell types, plays a crucial role in bone formation, growth, development, and fracture healing.^11^ Global Piezo1 deletion during skeletogenesis induces severe bone deformities and spontaneous fractures, while adult-stage Piezo1 silencing precipitates osteoporotic phenotypes.^12^ Our prior single-cell RNA sequencing (scRNA-seq) revealed pronounced Piezo1 enrichment in PSCs, osteoblast lineage cells, chondrocytes, and endothelial cells at the fracture site compared to intact bone tissue.^13^ Through comparative analysis of chondrocyte-specific Piezo1 knockout (*Piezo1*^*Col2a1*^) and floxed control (*Piezo1*^*f/f*^) murine fracture models, we demonstrated that *Piezo1*^*Col2a1*^ mice exhibited abnormal transcriptional states, stagnated at the hypertrophic cartilage stage, and had difficulty transdifferentiating into osteoblasts, as evidenced by scRNA-seq of callus tissues.^14^ Additionally, mitochondrial dysfunction was evident in chondrocytes with a Piezo1 knockout, which may contribute to the reduced osteogenic capacity of these cells following Piezo1-knockout.

Previous studies have demonstrated that Piezo1 activation in vascular endothelial cells stimulates adenosine triphosphate (ATP) production by enhancing mitochondrial respiration and glycolytic activity.^15^ Mitochondria are among the most important organelles in chondrocytes and are involved in ATP generation through oxidative phosphorylation.^16^ The maintenance of normal mitochondrial function is crucial for fracture healing. The impairment of mitochondrial function in chondrocytes during endochondral ossification blocks oxidative phosphorylation and significantly reduces ATP production.^17^ Our scRNA-seq analysis of day-14 fracture callus revealed pronounced upregulation of mitochondrial leucyl-tRNA synthetase 2 (Lars2) in *Piezo1*^*Col2a1*^ mice. This tRNA synthetase, localized to mitochondria, can affect mitochondrial function by influencing the translation of mitochondrial genes, which is an essential step in cell differentiation.^18^ However, the molecular mechanism linking Piezo1 and Lars2 remains unclear. β-catenin, a key intracellular signaling molecule in the Wnt/β-catenin pathway, plays a vital role in promoting osteogenesis.^19,20^ Our team has previously demonstrated that Piezo1 promotes osteogenic differentiation of PSCs by regulating β-catenin, thereby accelerating fracture healing.^13^ Hu et al. suggested that Piezo1 stimulates the proliferation and osteogenic differentiation of Gli1^+^ BMSCs through the activation of β-catenin and its target gene transcription factor 4 (ATF4), while inhibition of β-catenin expression significantly attenuated the effects of Yoda1 on bone metabolism.^21^ These collective findings establish β-catenin as the central signaling node through which Piezo1 orchestrates mitochondrial-osteogenic coupling.

In the current study, we first confirmed Piezo1 knockout induced chondrocytic mitochondrial bioenergetic impairment, compromised endochondral ossification progression, and identified Lars2 as a key downstream effector through scRNA-seq. Next, inhibition of high levels of Lars2 in chondrocytes alleviated mitochondrial dysfunction and promoted the endochondral ossification in fracture healing. Finally, we found the interaction between β-catenin and Lars2 proteins in chondrocytes and demonstrated that Piezo1 participates in fracture healing by regulating Lars2 through β-catenin-mediated signaling. Collectively, our findings point to a new perspective on the role of chondrocytes in endochondral ossification, which suggest that Piezo1/β-catenin/Lars2 signaling axis in chondrocytes may be a potential therapeutic target for accelerating fracture healing.

## Results

### Piezo1 deficiency inhibits its transdifferentiation into osteoblasts in ATDC5 cells

**Fig. 1:**
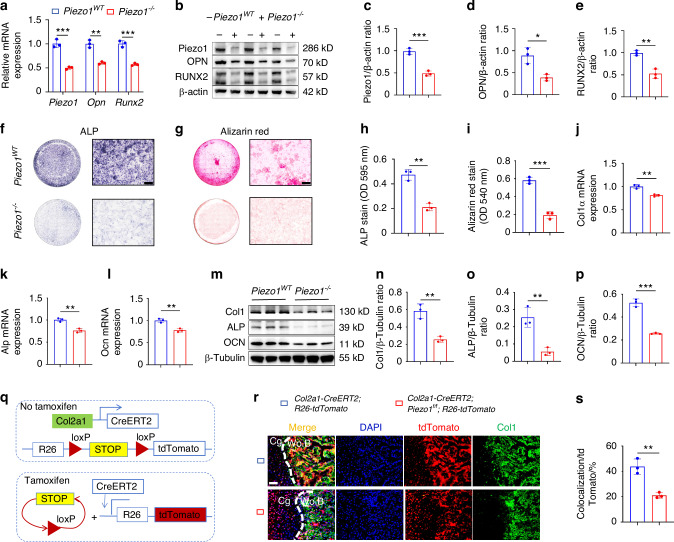
Knockout of the Piezo1 gene in chondrocytes inhibits their transdifferentiation into osteoblasts. **a** qPCR was used to detect changes in the expression of Piezo1 and osteogenic markers Opn (Spp1) and Runx2 in each group after osteogenic induction culture, *n* = 3. **b**–**e** WB was used to detect changes in the expression of Piezo1 and osteogenic markers OPN and RUNX2 in each group after osteogenic induction culture and statistical analysis, *n* = 3. **f**, **g** ALP and alizarin red staining were used to detect ALP levels after 7 days of osteogenic induction culture and the expression of calcium nodules after 21 days of induction culture, scale = 200 μm. **h**, **i** Statistical analysis of ALP and Alizarin Red stain were performed, *n* = 3. **j**–**l** qPCR was used to detect changes in the expression of Col1α, Alp, and Ocn in each group, *n* = 3. **m**–**p** WB was used to detect changes in the expression of Col1α, ALP, and OCN in each group and statistical analysis, *n* = 3. **q** Schematic diagram of fluorescence colorimetric principle of gene mice *R26*-tdTomato. **r** Lineage tracing was used to detect the transdifferentiation forms of chondrocytes during endochondral ossification and the role of Piezo1 in endochondral ossification during fracture healing, scale = 50 μm, *n* = 3. Cg: Cartilage tissue; Wo.B: Woven bone tissue. blue: nucleus; red: tdTOmato; green: Col1; golden (colocalization): osteoblasts derived from chondrocytes’ transdifferentiation. **s** The statistical analysis of osteoblasts derived from chondrocytes’ transdifferentiation. *P < 0.05, **P < 0.01, and ***P < 0.001

To investigate the role of Piezo1 in endochondral ossification in vitro, we constructed an ATDC5 stable transgenic cell line with Piezo1 gene knockout (*Piezo1*^*–/–*^) using CRISPR-Cas9 gene editing. We then performed in vitro osteogenic differentiation cultures of both *Piezo1*^*WT*^ and *Piezo1*^*–/–*^ ATDC5 cells. A significant reduction in both mRNA and protein levels of Piezo1 in *Piezo1*^*–/–*^ ATDC5 cells compared with *Piezo1*^*WT*^ ATDC5 cells was revealed after quantitative polymerase chain reaction (qPCR) and Western blotting (WB) (Fig. 1a–c), confirming the successful generation of a stable *Piezo1*^*–/–*^ ATDC5 cell line. Furthermore, qPCR and WB results demonstrated a marked decrease in the mRNA and protein levels of the osteogenesis-related markers osteopontin (OPN), RUNX2, collagen type I (Col1), alkaline phosphatase (ALP), and osteocalcin (OCN) following Piezo1 knockout (Fig. 1a, b, d, e, j–p). ALP staining revealed a substantial decrease in ALP expression in *Piezo1*^*–/–*^ ATDC5 cells (Fig. 1f, h). Similarly, alizarin red staining revealed a significant reduction in calcium nodule deposition in *Piezo1*^*–/–*^ ATDC5 cells compared to that in the *Piezo1*^*WT*^ group (Fig. 1g, i). Collectively, these results demonstrate that Piezo1 plays a crucial role in endochondral ossification in vitro, and its knockout inhibits the transdifferentiation of chondrocytes into osteoblasts.

### *Piezo1*^*Col2a1*^ mice reported impaired endochondral ossification and delayed fracture healing

**Fig. 2:**
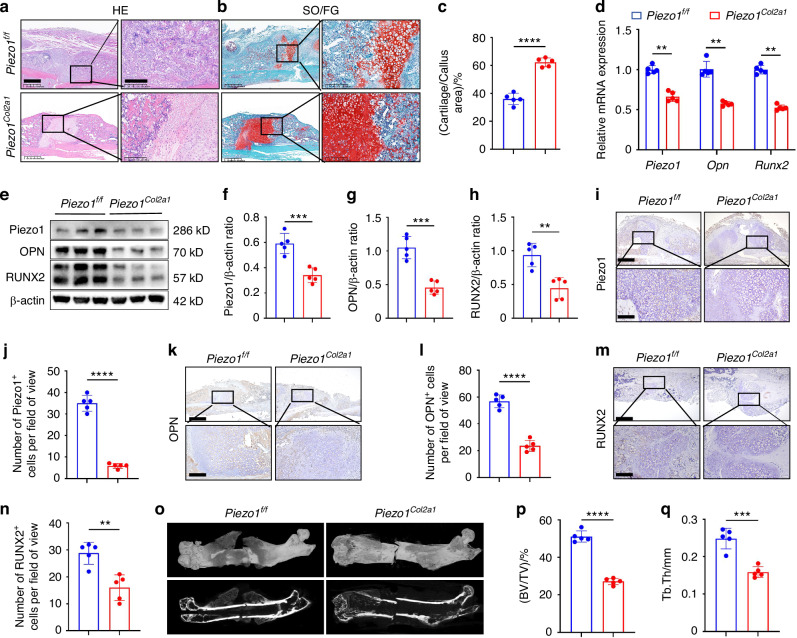
*Piezo1*^*Col2a1*^ mice have impaired endochondral ossification and delayed fracture healing. **a**, **b** HE and SO/FG staining were used to observe the morphological distribution of cartilage tissue and woven bone tissue in the callus of each group 14 days after femoral fracture, scale = 625 μm (left) and 200 μm (right). **c** The statistical analysis of the proportion of cartilage tissue to callus, *n* = 5. **d** qPCR was used to detect changes in the expression of Piezo1, Opn, and Runx2 in the callus of each group 14 days after femoral fracture. **e**–**h** WB was used to detect the expression changes of Piezo1, OPN, and RUNX2 in the callus of each group 14 days after femoral fracture, along with statistical analysis, *n* = 5. **i**, **j** IHC staining was used to detect the expression changes of Piezo1 in hypertrophic chondrocytes of callus in each group 14 days after femoral fracture, with statistical analysis, scale = 625 μm (up) and 200 μm (down), *n* = 5. **k**, **l** IHC staining was used to detect changes in the expression of OPN in hypertrophic chondrocytes of the callus in each group 14 days after femoral fracture, with statistical analysis, scale = 625 μm (up) and 200 μm (down), *n* = 5. **m**, **n** IHC staining was used to detect changes in the expression of RUNX2 in hypertrophic chondrocytes of the callus in each group 14 days after femoral fracture, with statistical analysis, scale = 625 μm (up) and 200 μm (down), *n* = 5. **o** Micro-CT observation of representative bone callus 3D reconstruction and coronal section images in each group 14 days after femoral fracture, *n* = 5. **p**, **q** BV/TV and Tb.Th analysis of callus 14 days after femoral fracture in each group, *n* = 5. **P < 0.01, ***P < 0.001, and ****P < 0.000 1

To investigate the transdifferentiation of chondrocytes during endochondral ossification and the role of Piezo1 in fracture healing, we conducted lineage tracing analysis on fracture models of wild type mice carrying *R26*-tdTomato fluorescent reporter gene in chondrocytes (*Col2a1*-CreERT2; *R26*-tdTomato) and *Piezo1*^*f/f*^ mice carrying *R26*-tdTomato fluorescent reporter gene in chondrocytes (*Col2a1*-CreERT2; *Piezo1*^*f/f*^; *R26*-tdTomato). The results reported that chondrocytes could transdifferentiate into osteoblasts during endochondral ossification, and the specific knockout of Piezo1 in chondrocytes inhibited their transdifferentiation into osteoblasts (Fig. 1q–s). Haematoxylin-eosin (HE) staining was performed to observe morphological changes in the callus of *Piezo1*^*f/f*^ and *Piezo1*^*Col2a1*^ mice. We found that the woven bone was mainly distributed at both ends, while cartilage tissue was localized in the center, indicating that the endochondral ossification process during fracture healing started at the ends of the fracture and gradually progressed toward the center (Fig. 2a). The morphological distribution and proportion of cartilage and woven bone tissue in the two groups of mice were assessed by Safranin O/Fast Green (SO/FG) staining. The results reported that the proportion of cartilage tissue was significantly higher in *Piezo1*^*Col2a1*^ mice than in *Piezo1*^*f/f*^ mice (Fig. 2b, c). Moreover, qPCR and WB analyses revealed that the mRNA and protein levels of Piezo1 were significantly reduced in the callus of *Piezo1*^*Col2a1*^ mice, confirming their successful generation of *Piezo1*^*Col2a1*^ mice (Fig. 2d–f). Additionally, the mRNA and protein levels of osteogenesis-related markers OPN and RUNX2, were also significantly reduced in the callus of *Piezo1*^*Col2a1*^ mice (Fig. 2d, e, g, h), indicating that the endochondral ossification is inhibited in *Piezo1*^*Col2a1*^ mice.

Similarly, immunohistochemistry (IHC) experiments revealed an increased proportion of HTCs in the callus of *Piezo1*^*Col2a1*^ mice compared with *Piezo1*^*f/f*^ mice. The expression of Piezo1 (Fig. 2i, j) and the osteogenic markers OPN (Fig. 2k, l) and RUNX2 (Fig. 2m, n) were significantly reduced in the chondrocytes. Furthermore, we performed micro-computed tomography (micro-CT) scans on both *Piezo1*^*f/f*^ and *Piezo1*^*Col2a1*^ mice 14 days (Fig. 2o), 21 days (Fig. S1p), and 28 days (Fig. S1p) after femoral fracture. The results showed that the fractures in *Piezo1*^*f/f*^ mice had essentially healed, and the callus tissue had largely completed remodeling by 28 days post-fracture (Fig. S1p). The results also depicted a significant reduction in bony callus tissue in the *Piezo1*^*Col2a1*^ mice. Since our focus was on the endochondral ossification process during fracture healing, we selected 14-day post-fracture mice for the study. Bone volume/total volume (BV/TV) (Fig. 2p), and trabecular thickness (Tb.Th) (Fig. 2q) values of callus tissue in *Piezo1*^*Col2a1*^ mice were significantly lower compared with *Piezo1*^*f/f*^ mice 14 days after femoral fracture. These findings suggest that Piezo1 plays a role in regulating endochondral ossification and, consequently, affects fracture healing.

### Piezo1 deficiency leads to mitochondrial dysfunction

**Fig. 3:**
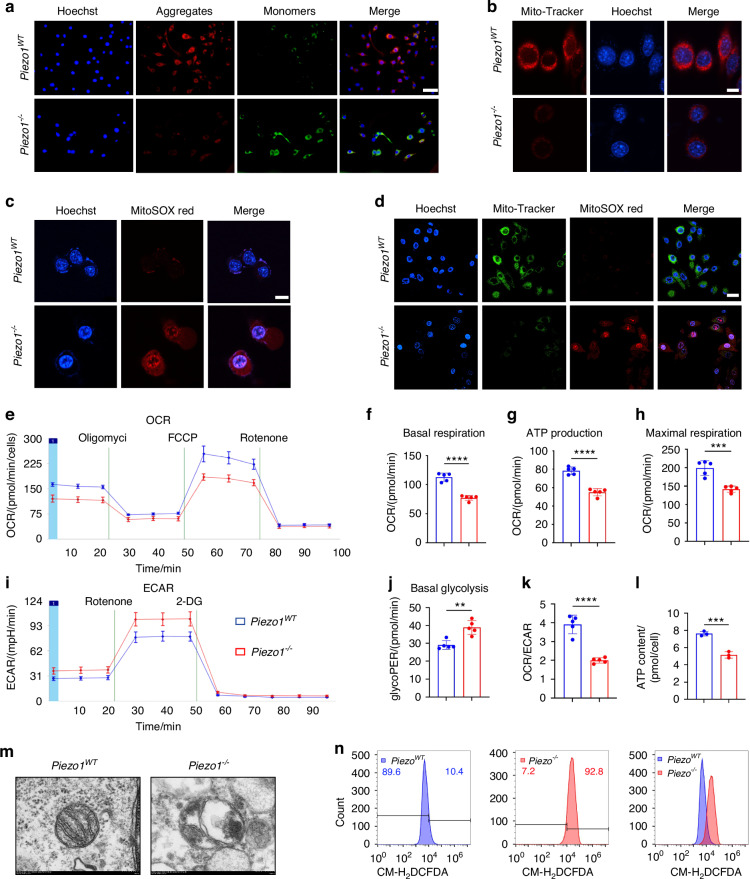
Knockout of Piezo1 gene in ATDC5 cells leads to mitochondrial dysfunction. **a** JC-1 staining was used to detect changes in mitochondrial membrane potential in each group, scale = 50 μm, *n* = 3. Blue fluorescence indicates the nuclei of live cells, red fluorescence indicates that JC-1 exists in the form of polymers (J-aggregates) in mitochondria, and green fluorescence indicates that JC-1 exists in the form of monomers in the mitochondria. **b** Mito-Tracker Red CMXRos staining was used to detect fluorescence changes in biologically active mitochondria in each group, scale = 2.51 μm, *n* = 3. Red fluorescence indicates mitochondria with biological activity, and blue fluorescence indicates the nuclei of live cells. **c** MitoSOX Red was used to detect changes in the expression of superoxide in the mitochondria of each group scale = 10 μm, *n* = 3. Blue fluorescence indicates the nuclei of live cells; red fluorescence represents the oxidation product formed by the reaction with MitoSOX Red. **d** Mito-Tracker Green and MitoSOX Red double staining was used to detect changes in mitochondrial function in each group scale = 20 μm, *n* = 3. Blue fluorescence indicates the nuclei of live cells, green fluorescence indicates mitochondrial morphology in live cells, and red fluorescence indicates the oxidation product formed by the reaction with MitoSOX Red. **e** The Agilent Seahorse XFe24 analyzer was used to measure the OCR of each group to characterize the mitochondrial function, *n* = 5. **f** The OCR of basal respiration, *n* = 5. **g** The OCR of ATP production, *n* = 5. **h** The OCR of maximal respiration, *n* = 5. **i** The Agilent Seahorse XFe24 analyzer was used to measure the ECAR of each group, *n* = 5. **j** The glycoPER in each group, *n* = 5. **k** The ratio of OCR/ECAR in each group, *n* = 5. **l** An ATP assay kit was used to detect ATP levels in each group, *n* = 3. **m** TEM was used to observe the mitochondrial microstructure and ultrafine details of the cells in each group, scale = 200 nm, *n* = 3. **n** ROS levels in each group were quantified by flow cytometry with CM-H_2_DCFDA staining, *n* = 3. **P < 0.01, ***P < 0.001, and ****P < 0.000 1

Mitochondria, among the most crucial organelles in chondrocytes, generate ATP through oxidative phosphorylation to provide energy during endochondral ossification.^22^ When mitochondrial function in chondrocytes is impaired, the oxidative phosphorylation process is disrupted, leading to a significant reduction in ATP production and hindering the transdifferentiation of chondrocytes into osteoblasts.^23^ To verify whether Piezo1 affects endochondral ossification by regulating mitochondrial function in chondrocytes, we performed a series of experiments. Mitochondrial membrane potential was assessed using JC-1 staining. Loss of Piezo1 resulted in a significant decrease in the mitochondrial membrane potential (Fig. 3a). Mito-Tracker Red CMXRos staining was used to specifically label bioactive mitochondria in cells. We observed clear and bright red fluorescence in the mitochondria of the *Piezo1*^*WT*^ ATDC5 cells. However, the red fluorescence in *Piezo1*^*–/–*^ ATDC5 cells decreased significantly, and the fluorescence was diffused (Fig. 3b). The mitochondrial superoxide assay using MitoSOX Red revealed unoxidized MitoSOX Red in *Piezo1*^*WT*^ ATDC5 cells with very weak red fluorescence. In contrast, *Piezo1*^*–/–*^ ATDC5 cells exhibited significantly enhanced red fluorescence in both the mitochondria and nucleus compared with *Piezo1*^*WT*^ cells (Fig. 3c). Moreover, we performed double staining with Mito-Tracker Green and MitoSOX Red to examine the mitochondrial changes. In *Piezo1*^*WT*^ cells, strong green fluorescence was observed, with minimal red fluorescence. However, in *Piezo1*^*–/–*^ ATDC5 cells, green fluorescence was significantly reduced, while red fluorescence was markedly increased (Fig. 3d). These findings indicate a significant decrease in active mitochondria and an increase in superoxide generation within the mitochondria following Piezo1 deletion.

To further assess changes in mitochondrial function, we measured the OCR and extracellular acidification aate (ECAR) in both *Piezo1*^*WT*^ and *Piezo1*^*–/–*^ cells. The results revealed a significant decrease in the overall OCR values and a rise in the overall ECAR values in *Piezo1*^–/–^ ATDC5 cells compared with *Piezo1*^*WT*^ cells (Fig. 3e, i). The reduced basal respiration OCR value in *Piezo1*^*–/–*^ ATDC5 cells suggests a decrease in the energy demand of these cells under basal conditions (Fig. 3f). The reduced ATP production in *Piezo1*^*–/–*^ ATDC5 cells indicated a diminished capacity for cellular ATP synthesis (Fig. 3g). And the decrease in the OCR during maximal respiration indicates a reduced capacity for cellular respiration, suggesting a decline in the ability of cells to reach their maximum respiratory potential (Fig. 3h). The rise of glycolytic proton efflux rate (glycoPER) in *Piezo1*^*–/–*^ ATDC5 cells indicated that the anaerobic respiration of cells increases (Fig. 3j). The decreased ratio of OCR/ECAR indicated that the *Piezo1*^*–/–*^ ATDC5 cells shift from aerobic respiration to anaerobic respiration, and the cellular energy metabolism declines (Fig. 3k). We also measured ATP levels using an ATP Assay Kit. The results revealed a significant decrease in ATP concentration in *Piezo1*^*–/–*^ ATDC5 cells compared to *Piezo1*^*WT*^ cells (Fig. 3l).

What’s more, we observed obvious morphological changes in *Piezo1*^*–/–*^ cells compared with *Piezo1*^*WT*^ cells through optical microscope (Fig. S1a). Then, we used transmission electron microscopy (TEM) to observe the ultrastructure of mitochondria. We found a significant reduction in mitochondrial cristae and enlargement of the mitochondrial matrix in *Piezo1*^*–/–*^ cells (Fig. 3m). And we found through the CCK-8 assay that the proliferation ability of *Piezo1*^*–/–*^ cells decreased significantly (Fig. S1b). Similarly, we found that the level of reactive oxygen species (ROS) in *Piezo1*^*–/–*^ cells increased significantly (Fig. 3n). Furthermore, qPCR and WB results demonstrated a marked changes in the mRNA and protein levels of the mitochondrial dynamics-related markers mitofusin-1 (Mfn1), mitofusin-2 (Mfn2), optic atrophy 1 (OPA1), and dynamin-related protein 1 (Drp1) following Piezo1 knockout (Fig. S1c-k). These results demonstrate that Piezo1 plays a critical role in maintaining the mitochondrial structure and function. In conclusion, our data suggest that Piezo1 deficiency leads to defects in the mitochondrial structure and function. When we used mitoquinone mesylate (MitoQ) to protect the mitochondrial function in *Piezo1*^*–/–*^ ATDC5 cells, ALP and alizarin red staining revealed that the osteogenic ability of the cells were enhanced (Fig. S1l-o). These results suggest that Piezo1 can regulate the transdifferentiation of chondrocytes into osteoblasts by influencing mitochondrial function.

### Piezo1 could regulate Lars2 expression both in vivo and in vitro

**Fig. 4:**
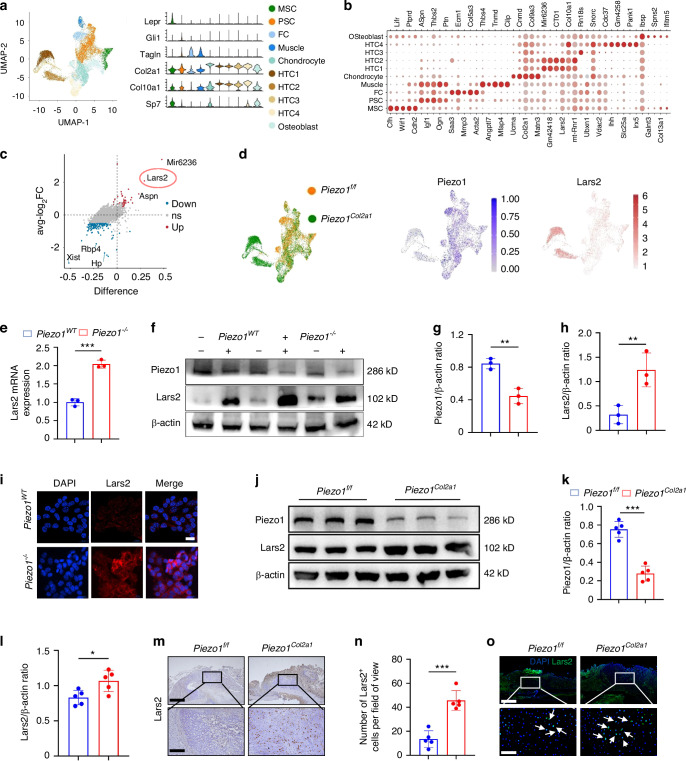
DEGs were screened using scRNA-seq, and the knockout of Piezo1 was verified to regulate Lars2 both in vivo and in vitro. **a** Cell grouping of calluses in scRNA-seq and cell identification based on specific gene expression. **b** The dot plot displays the specifically expressed genes of the main cell types isolated from the cell subpopulation. **c** DEGs in hypertrophic chondrocytes after specific knockout of Piezo1 in mouse chondrocytes. **d** Changes in Lars2 expression after specific Piezo1 knockout in mouse chondrocytes. **e** qPCR was performed to detect changes in Lars2 expression in each group, *n* = 3. **f**–**h** WB was used to detect changes in Piezo1 and Lars2 expression in each group and statistical analysis, *n* = 3. **i** IF staining was used to detect changes in Lars2 expression in each group, scale = 20 μm, *n* = 3. Blue fluorescence indicates the nucleus, and red fluorescence indicates the Lars2 protein. **j**–**l** WB was used to detect the expression changes of Piezo1 and Lars2 in the callus of each group after 14 days of femoral fracture, and statistical analysis, *n* = 5. **m**, **n** IHC staining was used to detect the expression changes of Lars2 in hypertrophic chondrocytes of callus in each group 14 days after femoral fracture and statistical analysis, scale = 625 μm (up) and 200 μm (down), *n* = 5. **o** IF staining was used to detect the expression level of Lars2 in hypertrophic chondrocytes of the callus in each group after 14 days of femoral fracture, scale = 625 μm (up) and 100 μm (down), *n* = 5. Blue fluorescence indicates the nucleus, and green fluorescence indicates Lars2 expression. White arrows: Chondrocytes expressing Lars2. *P < 0.05, **P < 0.01, and ***P < 0.001

To further explore how Piezo1 regulates chondrocyte mitochondrial function and affects the process of endochondral ossification in fracture healing, we reanalyzed scRNA-seq data from the callus tissues of *Piezo1*^*f/f*^ and *Piezo1*^*Col2a1*^ mice 14 days after femoral fracture.^14^ First, callus tissue from the scRNA-seq data was sorted based on specific gene expression profiles (Fig. 4a). The hypertrophic chondrocyte population, characterized by the high expression of *Col2a1* and Col10a1, was selected. Gene expression levels in the scRNA-seq data of callus tissue cells revealed increased expression of Lars2 in HTC1 and HTC2 subsets (Fig. 4b). To further explore the potential mechanism by which Piezo1 regulates chondrocyte-osteoblast transdifferentiation, we examined the differentially expressed genes (DEGs) in HTCs of *Piezo1*^*Col2a1*^ and *Piezo1*^*f/f*^ groups and found that Lars2 was significantly upregulated in hypertrophic chondrocytes after Piezo1 deficiency (Fig. 4c, d).

Lars2, a gene associated with mitochondrial function, plays a crucial role in maintaining mitochondrial function by regulating the translation of mitochondrial genes that are essential for cell differentiation. Through the scRNA-seq analysis mentioned earlier, we observed an increased expression of Lars2 in chondrocytes from *Piezo1*^*Col2a1*^ mice. To test this hypothesis, we performed a series of in vivo and in vitro experiments. qPCR and WB analyses revealed that both the mRNA and protein levels of Lars2 were significantly elevated in *Piezo1*^*–/–*^ ATDC5 cells compared with the *Piezo1*^*WT*^ group (Fig. 4e–h). Moreover, immunofluorescence (IF) staining further confirmed the markedly increased expression of Lars2 in *Piezo1*^*–/–*^ ATDC5 cells (Fig. 4i). These results are consistent with in vivo observations. In the callus tissue of *Piezo1*^*Col2a1*^ fracture mice, Lars2 protein levels were also significantly higher than those in *Piezo1*^*f/f*^ fracture mice (Fig. 4j, l). Furthermore, IHC and IF experiments revealed significantly increased Lars2 expression in hypertrophic chondrocytes of callus tissue from *Piezo1*^*Col2a1*^ mice (Fig. 4m–o). Collectively, these findings suggest that Piezo1 knockout leads to a significant increase in Lars2 levels both in vitro and in vivo.

### Inhibition of Lars2 ameliorated the mitochondrial dysfunction and promoted their transdifferentiation into osteoblasts in *Piezo1*^*–/–*^ ATDC5 cells

To investigate whether Lars2 acts as a key downstream regulator of Piezo1, we constructed Lars2 interference plasmids (shRNAs) and a negative control plasmid (NC) for in vitro experiments. Three interference sequences targeting Lars2 were designed (Table S1) and incorporated into the U6-MCS-CAG-EGFP vector to construct corresponding plasmids (shLars2-1, shLars2-2, and shLars2-3). These plasmids were then transfected into both *Piezo1*^*WT*^ and *Piezo1*^*–/–*^ ATDC5 cells. The results indicated that shLars2-1 plasmid demonstrated the most effective interference (Fig. S2a, b). Therefore, shLars2-1 interference plasmid was selected for subsequent studies.

**Fig. 5:**
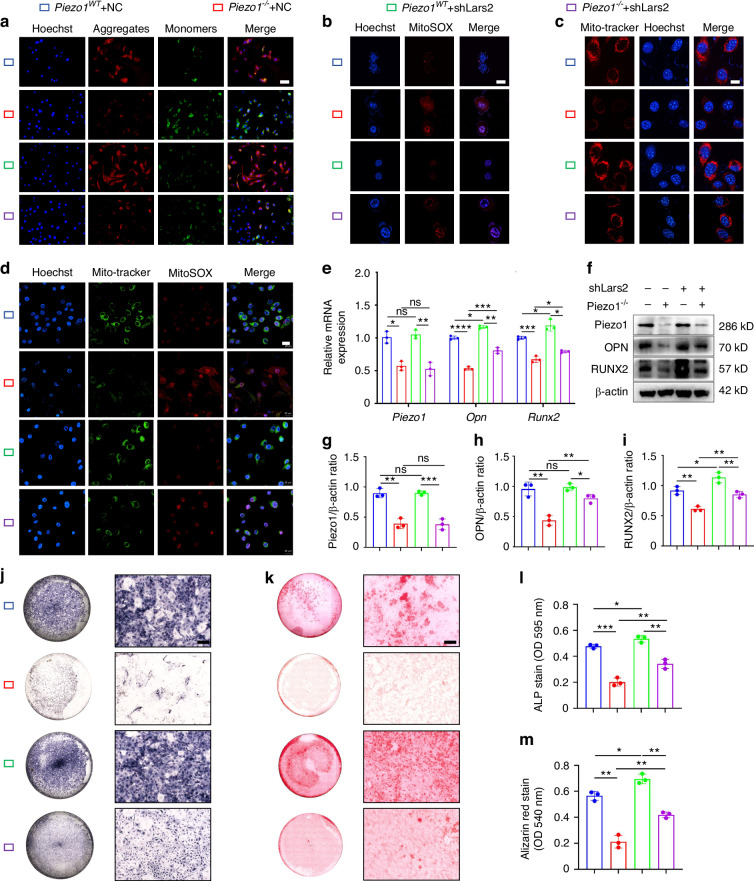
Inhibition of Lars2 ameliorated mitochondrial dysfunction in *Piezo1*^*–/–*^ ATDC5 cells and promoted their transdifferentiation into osteoblasts. **a** JC-1 staining was used to detect changes in the mitochondrial membrane potential in each group, scale = 50 μm, *n* = 3. Blue fluorescence indicates the nuclei of live cells, red fluorescence represents JC-1 polymers (J-aggregates) in the mitochondria, and green fluorescence represents JC-1 monomers in the mitochondria. **b** MitoSOX Red staining was used to detect changes in superoxide expression within the cell mitochondria in each group, scale = 10 μm, *n* = 3. Blue fluorescence indicates the nuclei of live cells, and red fluorescence represents the oxidation product formed by the reaction with MitoSOX Red. **c** Mito-Tracker Red CMXRos staining was used to detect fluorescence changes in mitochondria with biological activity in the cells of each group, scale = 2.51 μm, *n* = 3. Red fluorescence indicates mitochondria with biological activity, and blue fluorescence indicates the nuclei of live cells. **d** Mito-Tracker Green and MitoSOX Red double staining were used to detect changes in mitochondrial function in the cells of each group, scale = 20 μm, *n* = 3. Blue fluorescence indicates the nuclei of live cells, green fluorescence represents mitochondrial morphology, and red fluorescence represents the oxidation product formed by reaction with MitoSOX Red. **e** qPCR was used to detect the expression of Piezo1, Opn, and Runx2 in each group, *n* = 3. **f**–**i** WB was used to detect the expression of Piezo1, OPN, and RUNX2 in each group, and statistical analysis was performed, *n* = 3. **j**, **k** ALP and alizarin red staining were used to detect ALP levels after 7 days of osteogenic induction culture and the expression of calcium nodules after 21 days of induction culture, scale = 200 μm. **l**, **m** Statistical analysis of ALP and alizarin red stain were performed, *n* = 3. *P < 0.05, **P < 0.01, ***P < 0.001, ****P < 0.000 1, and ns > 0.05

Lars2 knockdown increased mitochondrial membrane potential and activity and decreased mitochondrial superoxide synthesis in *Piezo1*^*–/–*^ ATDC5 cells, as measured by JC-1 staining (Fig. 5a), and MitoSOX Red staining (Fig. 5b), Mito-Tracker Red CMXROS staining (Fig. 5c), and Mito-Tracker Green with MitoSOX Red double staining (Fig. 5d). And inhibition of elevated Lars2 levels in *Piezo1*^*–/–*^ ATDC5 cells modulated mitochondrial dynamics-related markers (Mfn1, Mfn2, OPA1, and Drp1), and then restored mitochondrial functional homeostasis (Fig. S2j–r). Furthermore, inhibition of Lars2 rescued impaired chondrocyte endochondral ossification in *Piezo1*^*–/–*^ ATDC5 cells, as assessed by increased osteogenic markers (Fig. 5e–i; Fig. S2c–i). The ALP and alizarin red staining results support these findings (Fig. 5j–m). Accordingly, Piezo1 regulates mitochondrial function and endochondral ossification in ATDC5 cells via the Lars2.

### Inhibition of Lars2 accelerated fracture healing in *Piezo1*^*Col2a1*^ mice

**Fig. 6:**
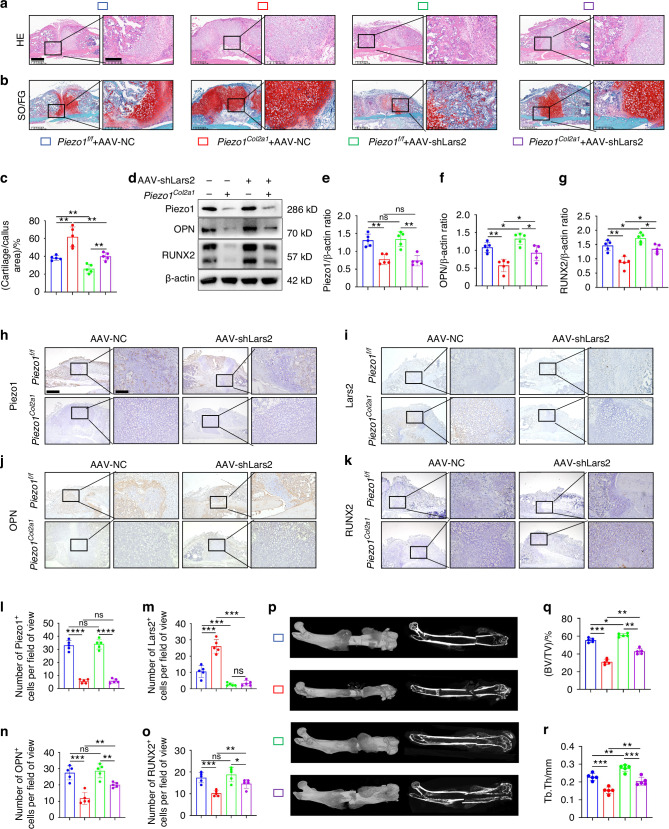
Inhibition of Lars2 in the chondrocytes of the callus promotes fracture healing in *Piezo1*^*Col2a1*^ mice. **a**, **b** HE and SO/FG staining were used to observe the morphological distribution of cartilage tissue and woven bone tissue in the callus of each group 14 days after femoral fracture, scale = 625 μm (left) and 200 μm (right). **c** The statistical analysis of the proportion of cartilage tissue to callus, *n* = 5. **d**–**g** WB was used to detect the expression changes of Piezo1, OPN, and RUNX2 in the callus of each group 14 days after femoral fracture, along with statistical analysis, *n* = 5. **h**–**o** IHC staining were used to detect the expression changes of Piezo1, Lars2, OPN, and RUNX2 in hypertrophic chondrocytes of callus in each group 14 days after femoral fracture, and statistical analysis, scale = 625 μm (up) and 200 μm (down), *n* = 5. **p** Micro-CT observation of representative bone callus 3D reconstruction and coronal section images, *n* = 5. **q**, **r** Analysis of BV/TV and Tb.Th of callus 14 days after femoral fracture in each group of mice, *n* = 5. *P < 0.05, **P < 0.01, ***P < 0.001, ****P < 0.000 1, and ns > 0.05

To investigate whether Piezo1 influences endochondral ossification in fracture healing by regulating Lars2 in the callus, we constructed an adeno-associated virus (AAV) carrying the shLars2-1 interference plasmid incorporated into the *Col2a1*p-EGFP-mir155 (MCS)-SV40 PolyA vector to specifically target chondrocytes in the callus and inhibit Lars2 expression. Morphological changes in the callus of each group were examined by HE staining (Fig. 6a). The proportion of cartilage tissue in the callus and the degree of fracture healing varied among the groups. SO/FG staining and statistical analysis were performed to determine the proportion of chondrocytes in the callus (Fig. 6b, c). The results reported that the proportion of chondrocytes in the callus of the *Piezo1*^*Col2a1*^ + AAV-NC group was significantly higher than that of the *Piezo1*^*f/f*^ + AAV-NC group, indicating that specific knockout of the Piezo1 gene in chondrocytes inhibits endochondral ossification and delays fracture healing. Furthermore, the proportion of chondrocytes in the *Piezo1*^*Col2a1*^ + AAV-shLars2 group was significantly lower than that in the *Piezo1*^*Col2a1*^ + AAV-NC group, indicating that the inhibition of elevated levels of Lars2 in chondrocytes promotes endochondral ossification and accelerates fracture healing.

WB analysis was used to assess Piezo1 and osteogenic markers, OPN and RUNX2, in callus tissue 14 days after femoral fracture (Fig. 6d–g). The results reported that shLars2-AAV significantly increased the expression of OPN and RUNX2 in *Piezo1*^*Col2a1*^ mice (Fig. 6f, g). Moreover, the expression of Piezo1, Lars2, and the osteogenic markers OPN and RUNX2 in femoral fractures was examined in each treated group by IHC staining (Fig. 6h–o). The findings revealed that shLars2-AAV treatment accelerated fracture healing in *Piezo1*^*Col2a1*^ mice. These results suggest that inhibiting Lars2 reverses the osteogenic impairment induced by Piezo1 deletion in chondrocytes and consequently restores endochondral ossification.

Finally, we performed micro-CT scans of the mouse specimens 14 days after the femoral fracture in each group, providing representative 3D reconstructions of the callus and coronal section images. The results demonstrated that compared with the *Piezo1*^*f/f*^ + AAV-NC group, the *Piezo1*^*Col2a1*^ + AAV-NC group exhibited significantly reduced bone callus, clear fracture ends, and less woven bone coverage (Fig. 6p). Moreover, a significant decrease in BV/TV and Tb.Th of the callus was observed, indicating that a specific knockout of Piezo1 in chondrocytes inhibits fracture healing (Fig. 6q, r). Compared with *Piezo1*^*Col2a1*^ + AAV-NC group, *Piezo1*^*Col2a1*^ + AAV-shLars2 group exhibited increased bone callus with trabecular coverage at the fracture ends (Fig. 6p), along with an increase in BV/TV and Tb.Th (Fig. 6q, r). These results suggest that the inhibition of Lars2 can reverse the delay in fracture healing caused by Piezo1 knockout, highlighting that Piezo1 affects endochondral ossification in fracture healing by regulating Lars2 in chondrocytes.

### Piezo1 regulated Lars2 through β-catenin signaling pathway

**Fig. 7:**
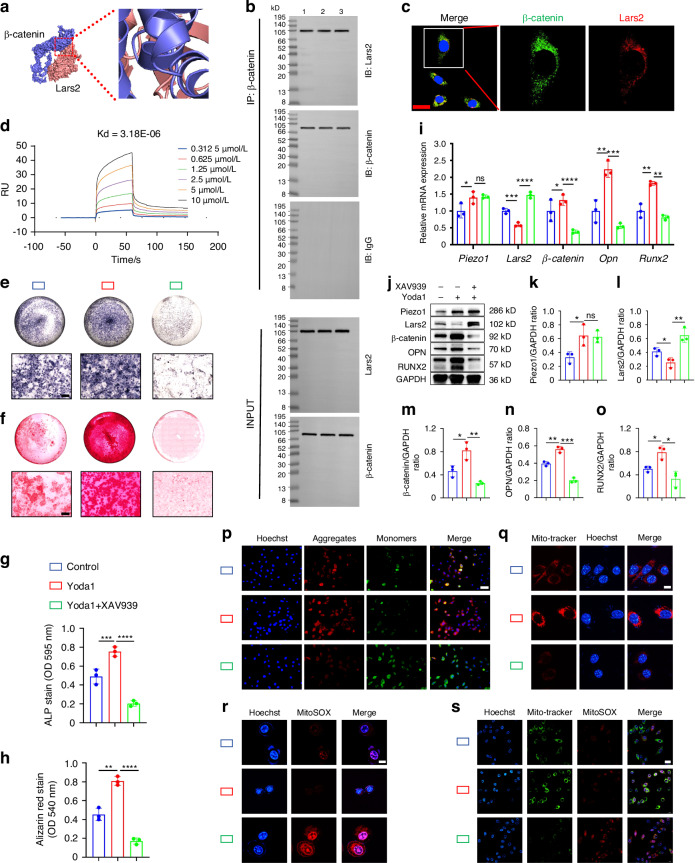
Piezo1 can regulate Lars2 to affect mitochondrial function and promote endochondral ossification through β-catenin signaling. **a** The protein structures of CTNNB1 (β-catenin) and Lars2 were downloaded from the UniProt database, and the interaction between β-catenin and Lars2 was studied using Hdock. Blue: β-catenin protein structure; pink: Lars2 protein structure. **b** COIP analysis of the interaction between β-catenin and Lars2 proteins. **c** Immunofluorescence co-staining was performed to detect the co-localization between β-catenin and Lars2 proteins, scale = 20 µm. Blue fluorescence: cell nucleus; green fluorescence: β-catenin; red fluorescence: Lars2. **d** SPR experiments were performed to analyze the binding affinity between β-catenin and Lars2 protein. **e**, **f** ALP and alizarin red staining were used to detect ALP levels after 7 days of osteogenic induction culture and the expression of calcium nodules after 21 days of induction culture, scale = 200 μm. **g**, **h** Statistical analysis of ALP and alizarin red were performed, *n* = 3. **i** qPCR was used to detect mRNA expression of Piezo1, β-catenin, Lars2, Opn, and Runx2 in each group, *n* = 3. **j**–**o** WB was used to detect the protein expression of Piezo1, β-catenin, Lars2, and the osteogenic markers OPN and RUNX2 in each group and statistical analysis, *n* = 3. **p** JC-1 staining was used to detect changes in mitochondrial membrane potential in each group, scale = 50 µm, *n* = 3. Blue fluorescence: nuclei of live cells; red fluorescence: JC-1 exists in the form of polymers (J-aggregates) in mitochondria; green fluorescence: JC-1 exists in the form of monomers in mitochondria. **q** Mito-Tracker Red CMXRos staining was used to detect fluorescence changes in mitochondria with biological activity in each group, scale = 2.51 µm, *n* = 3. Red fluorescence: Mitochondria with biological activity; blue fluorescence: Nuclei of live cells. **r** MitoSOX Red was used to detect changes in superoxide expression within cell mitochondria in each group, scale = 10 µm, *n* = 3. Blue fluorescence: nuclei of live cells; red fluorescence: oxidation product formed by reaction with MitoSOX Red. **s** Mito-Tracker Green and MitoSOX Red double staining were used to detect changes in mitochondrial function in each group, scale = 20 µm, *n* = 3. Blue fluorescence: nuclei of live cells; green fluorescence: mitochondrial morphology in live cells; red fluorescence: oxidation product formed by reaction with MitoSOX Red. *P < 0.05, **P < 0.01, ***P < 0.001, ****P < 0.000 1, and ns > 0.05

Next, we explored the regulatory mechanisms underlying Piezo1 and Lars2 expression. β-catenin, a key regulator of osteogenic differentiation, fracture repair, and osteoporosis, plays a crucial role in these processes.^24^ Recent studies have indicated that Piezo1 promotes osteogenesis through the Wnt/β-catenin signaling pathway and accelerates bone defect healing by enhancing the coupling of osteogenesis and angiogenesis.^25^ To investigate whether β-catenin interacts with Lars2 in the context of Piezo1 regulation, we retrieved the protein structures of CTNNB1 (β-catenin) and Lars2 from the UniProt database and studied their interactions using Hdock. The docking results revealed a binding energy of –269.07 kcal/mol between β-catenin and Lars2 (Fig. 7a). Residues around the protein-protein interaction interface were found to form hydrogen bonds, stabilizing the protein-protein complex. Pymol 2.3.0 analysis of the docking model demonstrated that PHE-560, ARG-550, ARG-542, GLN-601, and ASN-594 of β-catenin formed hydrogen bonds with GLU-837, GLU-831, ASP-829, and GLN-715 of Lars2. These hydrogen bonds have lengths of 3.3, 2.2, 3.0, 2.9, and 2.9 Å, respectively (Fig. S4). This suggests a potential interaction between β-catenin and Lars2. To confirm this, we performed immunoprecipitation experiments to verify the interaction between β-catenin and Lars2 (Fig. 7b). We also demonstrated co-localization between β-catenin and Lars2 proteins through immunofluorescence co-staining experiments (Fig. 7c). Furthermore, we conducted surface plasmon resonance (SPR) experiments using the purified β-catenin and Lars2 proteins. The results showed that β-catenin bound to Lars2 with a kinetic affinity constant (KD) value of 3.18 × 10^−6^, demonstrating a moderate interaction between the two proteins (Fig. 7d).

To further explore the mechanism by which Piezo1 regulates Lars2 expression, we used the Piezo1 agonist Yoda1 and β-catenin signaling inhibitor XAV939. qPCR and WB analyses revealed that in the Yoda1-treated group of ATDC5 cells, the expression of both Piezo1 and β-catenin was significantly increased, while Lars2 expression was significantly decreased compared with the control group. This suggests that the activation of Piezo1 promotes β-catenin expression and inhibits Lars2 expression (Fig. 7i–m). In contrast, in the Yoda1 + XAV939 co-treatment group, Piezo1 expression remained unchanged, β-catenin protein levels were reduced, and Lars2 expression significantly increased (Fig. 7i–m). These results indicate that Piezo1 regulates Lars2 expression through the β-catenin signaling pathway.

To investigate whether Piezo1 regulates cellular mitochondrial function through the β-catenin/Lars2 pathway, we examined the mitochondrial parameters in ATDC5 cells. Compared with the control group, the Yoda1-treated group exhibited increased mitochondrial membrane potential and activity along with decreased superoxide generation (Fig. 7p–s). Conversely, inhibition of β-catenin signaling with XAV939 resulted in decreased mitochondrial membrane potential and activity and increased superoxide production in ATDC5 cells (Fig. 7p–s). Additionally, we found that activation of Piezo1 regulates the expression of mitochondrial dynamics-related markers Mfn1, Mfn2, OPA1, and Drp1 through the β-catenin signaling pathway (Fig. S3h–p). These findings suggest that Piezo1 regulates Lars2 expression through the β-catenin signaling pathway, thereby influencing mitochondrial function.

To verify the molecular mechanism through which Piezo1 affects the transdifferentiation of chondrocytes into osteoblasts by regulating Lars2 expression by β-catenin signaling, we conducted a series of correlation experiments. qPCR and WB results revealed that in the Yoda1-treated group, Piezo1 expression was significantly higher than that in the control group, and the expression of osteogenic markers OPN, RUNX2, Col1, ALP, and OCN were also markedly elevated, indicating that Piezo1 activation promotes endochondral ossification (Fig. 7i–o; Fig. S3a–g). However, in the Yoda1 + XAV939 group, Piezo1 expression remained unchanged, β-catenin expression was reduced, Lars2 expression was increased, and the levels of osteogenic markers were significantly decreased. These results suggest that Piezo1 regulates Lars2 expression through β-catenin signaling, thereby affecting endochondral ossification. Furthermore, ALP and alizarin red staining confirmed these findings (Fig. 7e–h). Taken together, these data demonstrate that activation of Piezo1 inhibits Lars2 expression through β-catenin signaling, promoting endochondral ossification in vitro.

**Fig. 8:**
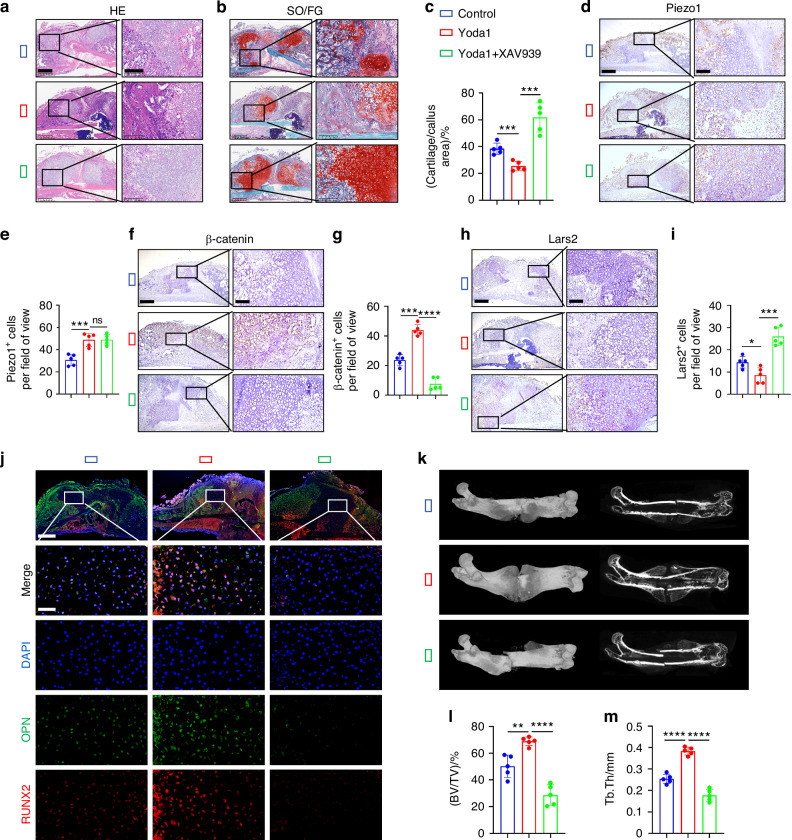
Piezo1 promotes endochondral ossification in vivo and accelerates fracture healing by modulating Lars2 through β-catenin signaling molecule. **a**, **b** HE and SO/FG staining were used to observe the morphological distribution of cartilage tissue and woven bone tissue in the callus of each group 14 days after femoral fracture, scale = 625 μm (left) and 200 μm (right). **c** The statistical analysis of the proportion of cartilage tissue to callus, *n* = 5. **d**–**i** IHC staining were used to detect the expression changes of Piezo1, β-catenin, and Lars2 in hypertrophic chondrocytes of callus in each group 14 days after femoral fracture, and statistical analysis, scale = 625 μm (up) and 200 μm (down), *n* = 5. **j** IF staining was used to detect the expression levels of osteogenic markers OPN and RUNX2 in hypertrophic chondrocytes of callus in each group 14 days after femoral fracture, scale = 625 μm (up) and 100 μm (down), *n* = 5. Blue fluorescence: Nucleus; green fluorescence: OPN expression level; red fluorescence: RUNX2 expression level. **k** Micro-CT observation of representative bone callus 3D reconstruction and coronal section images, *n* = 5. **l**, **m** Analysis of BV/TV and Tb.Th of callus 14 days after femoral fracture in each group of mice, *n* = 5. *P < 0.05, **P < 0.01, ***P < 0.001, ****P < 0.000 1, and ns > 0.05

To investigate whether Piezo1 could promote fracture healing through the regulation of Lars2 via the β-catenin signaling pathway, we characterized the callus in femur fractures 14 days post-injury across different experimental groups. First, we examined the morphological changes in the callus using HE staining and observed differences in the proportion of cartilage tissue among the groups (Fig. 8a), suggesting varying degrees of healing. The proportion of chondrocytes in the callus was further assessed using SO/FG staining and statistical analysis (Fig. 8b, c). The results reported that compared with the control group, the Yoda1 group exhibited a significantly reduced proportion of chondrocytes and an increased amount of woven bone, indicating a faster endochondral ossification process and more efficient fracture healing. Conversely, the Yoda1 + XAV939 group displayed a significant increase in the proportion of chondrocytes and a decrease in the woven bone in the callus, reflecting impaired endochondral ossification and delayed fracture healing. These findings suggest that activation of Piezo1 accelerates endochondral ossification, whereas inhibition of the Wnt/β-catenin signaling pathway hampers this process.

To further investigate the molecular mechanisms involved, we conducted IHC and tissue IF experiments. Compared with the control group, activation of Piezo1 using Yoda1 significantly increased Piezo1 expression in chondrocytes within the callus (Fig. 8d, e). Moreover, the β-catenin levels were significantly elevated (Fig. 8f, g), whereas Lars2 expression was significantly reduced (Fig. 8h, i). Moreover, osteogenic markers such as OPN and RUNX2 demonstrated notable increases (Fig. 8j). Conversely, when the Wnt/β-catenin signaling pathway was inhibited by XAV939, there were no significant changes in Piezo1 expression in chondrocytes of the callus (Fig. 8d, e). However, β-catenin levels were significantly reduced (Fig. 8f, g), Lars2 expression was significantly increased (Fig. 8h, i), and the osteogenic markers, OPN and RUNX2, were significantly reduced (Fig. 8j). These results confirmed that Piezo1 promotes endochondral ossification by regulating Lars2 expression through the β-catenin signaling pathway.

Finally, we performed micro-CT scans of the mouse specimens 14 days after femoral fracture in each group, which provided representative 3D reconstructions of the callus and coronal section images. The results demonstrated that compared with the control group, activation of Piezo1 using Yoda1 significantly increased the bone callus in mice, blurred the fracture ends, and enhanced the coverage of newly woven bone (Fig. 8k). Additionally, there was a significant increase in BV/TV and callus Tb. Th (Fig. 8l, m), indicating that activation of Piezo1 accelerated the fracture healing process. In contrast, in the Yoda1 + XAV939 group, we observed a decrease in the bone callus, clearer fracture ends, and significantly reduced coverage of the newly woven bone (Fig. 8k), indicating that blocking the Wnt/β-catenin signaling pathway inhibited the osteogenic activity of Piezo1. In summary, this study suggests that Piezo1 regulates Lars2 through the β-catenin signaling pathway, promoting endochondral ossification and accelerating fracture healing in vivo.

## Discussion

Endochondral ossification is critical in cases of instability, defects, and malreduction, playing a pivotal role in fracture healing.^26^ During this process, chondrocytes undergo rapid proliferation, maturation, and hypertrophy, forming cartilage crusts that bridge the fracture ends and stabilize the fracture defect.^27^ Recent studies have revealed that chondrocytes can also arise from PSCs and possess the ability to transdifferentiate into other cell types.^9^ Lineage tracing revealed that chondrocytes could transdifferentiate into osteoblasts during endochondral ossification, while the specific knockout of Piezo1 in chondrocytes inhibited this transdifferentiation. Moreover, indian hedgehog (IHH) signaling also plays important roles in endochondral ossification.^28^ In the mouse cartilage growth plate, IHH is expressed by chondrocytes, forming a negative feedback loop with parathyroid hormone-related peptide (PTHrP) and regulating chondrocyte to osteoblasts transdifferentiation. Bone morphogenetic protein (BMP) signaling has important regulatory roles in endochondral ossification.^29^ Chondrocytes and active osteoblasts all express BMP2 in human fracture callus, while BMP2 in chondrocytes decreases significantly when fracture nonunion occurs.^30^ Specific chondrocytes BMP2-deficient mice results in spontaneous fractures and failure to initiate the healing process. The classical Wnt/β-catenin signaling pathway is the key pathway regulating the interaction between chondrocytes and surrounding osteoblasts.^31^ Previous studies have demonstrated that inactivation of Wnt/β-catenin signaling inhibited chondrocyte hypertrophy and osteogenesis, while activation of it promoted osteoblastic differentiation of chondrocytes through BMP2.^32^ These works have helped to broaden the understanding of chondrocyte multifaceted roles in fracture healing.

Mechanical transduction, which converts mechanical stimuli into biological signals, plays a crucial role in fracture repairs. This process initiates a cascade of downstream signaling events that alter the metabolic activity of chondrocytes, ultimately influencing the phenotypic transitions of cells involved in endochondral ossification.^33,34^ A better understanding of mechanical signal transduction mechanisms in chondrocytes during fracture healing could open new therapeutic avenues for improving fracture repair. Piezo1, a recently discovered mechanosensitive calcium ion (Ca^2+^) channel protein, plays a critical role in biomechanical transduction in various cell types.^35^ It is encoded by the FAM38A gene and comprises three curved ‘leaves’ forming a central pore.^36^ Mechanical stimuli are transmitted through the deformation of the Piezo1 structure, which opens the channel and mediates Ca^2+^ influx into the cell.^37,38^ Studies have reported that Piezo1 is expressed in chondrocytes across various species, including humans, pigs, and mice, and plays a significant role in regulating bone development and homeostasis.^39^ Brylka et al. revealed that chondrocyte-specific Piezo1 ablation (*Piezo1*^*Col2a1Cre*^) causes severe postnatal skeletal defects, including near-complete trabecular bone loss beneath growth plates and spontaneous rib fractures localized to chondro-osseous junctions in 7-day-old mice.^40^ These findings establish Piezo1 as a master regulator of both physiological and pathological endochondral ossification, with therapeutic implications for targeting Piezo1 signaling in osteoarthritis-associated osteophyte formation.^40^ Paradoxically, despite its recognized regulatory importance, the precise mechanistic contributions of Piezo1 to chondrocyte-mediated fracture repair remain poorly defined. Our team previously performed scRNA-seq to delineate cellular dynamics during fracture repair, revealing stage-specific Piezo1 expression patterns across regenerative cell populations.^13^ The analysis revealed that Piezo1 was highly expressed in chondrocytes, suggesting that it may regulate the differentiation process. These findings led to the speculation that Piezo1 could serve as a key molecule for sensing mechanical stimuli during fracture repair. Further experimental research on the molecular mechanisms underlying Piezo1-mediated fracture healing is expected to provide new directions for fracture treatment. Based on this, we detected callus samples from *Piezo1*^*Col2a1*^ and *Piezo1*^*f/f*^ mice 14 days after fracture using scRNA-seq and screened for DEGs.^14^ Through integrated in vivo and in vitro validation, we established that Piezo1 governs endochondral ossification by modulating Lars2-mediated mitochondrial bioenergetics, mechanistically linking mechanical sensing to osteogenic commitment.

Lars2 is mainly located in mitochondria and plays a crucial role in regulating mitochondrial function by influencing mitochondrial gene translation processes, which are essential for cell differentiation.^41^ In myoblasts, the abnormal expression of Lars2 can significantly reduce cellular ATP levels and impair maximal and basal respiration, as indicated by a decrease in OCR.^18^ In patients with colorectal cancer, elevated levels of Lars2 lead to significant abnormalities in mitochondrial metabolism, particularly in NAD^+^ regeneration. Similarly, abnormal expression of Lars2 disrupts mitochondrial structure, causing mitochondrial fragmentation and significantly reducing the cross-sectional area of myoblasts.^18^ Mitochondria are essential organelles involved in complex energy metabolism processes and play crucial roles in cell survival and execution of cellular functions.^42^ The differentiation of bone progenitor cells into osteoblasts is closely linked to the activation of mitochondrial oxidative phosphorylation (OxPhos). Stimulation of mitochondrial OxPhos has been reported to enhance bone synthesis metabolism and promote fracture repair.^43^ In this study, we focused on the role of Lars2 in chondrocytes. Our results revealed that an abnormal increase in Lars2 levels caused a decrease in the mitochondrial membrane potential, weakened mitochondrial activity, and elevated mitochondrial superoxide levels in chondrocytes. These alterations can lead to mitochondrial dysfunction, reduced ATP production, and impaired endochondral ossification. This provides a new intervention target for the clinical prevention and treatment of delayed union and non-union fractures.

Hu et al. demonstrated that Piezo1 enhances the proliferation and osteogenic differentiation of Gli1^+^ BMSCs by activating β-catenin and its downstream target gene, ATF4. They proposed a molecular mechanism in which the Piezo1/Wnt/β-catenin/ATF4 signaling axis plays a crucial role in regulating the osteogenic differentiation of BMSCs.^21^ Guan et al. demonstrated that the Piezo1/β-catenin signaling pathway accelerates bone defect reconstruction and repair by promoting well-coordinated coupling of osteogenesis and angiogenesis.^25^ Jiang et al. found that, under stress conditions, Piezo1 modulates osteogenic differentiation and osteoclast activity through the activation of Wnt/β-catenin, thereby facilitating alveolar bone remodeling.^44^ These studies highlight the critical role of the Wnt/β-catenin pathway in regulating various cells and promoting osteogenesis. In this study, we explored the regulatory effects of the Wnt/β-catenin pathway on chondrocytes. We found that the inhibition of the Wnt/β-catenin signaling pathway reduced the regulatory effect of Piezo1 on Lars2, which subsequently affected mitochondrial function and impaired endochondral ossification. Our results further demonstrate a new mechanism by which the Piezo1/β-catenin/Lars2 signaling axis regulates mitochondrial function in chondrocytes, thereby influencing fracture healing.

This study has some limitations. First, owing to ethical constraints, human callus tissue samples were not available, and in vivo experimental data were derived from mice. Given the species differences between humans and mice, future studies should consider using primates to validate the conclusions of this study. Second, the in vitro experiments used *Piezo1*^*WT*^ and *Piezo1*^*–/–*^ ATDC5 cells but did not involve primary chondrocytes from mice. In subsequent studies, we plan to extract *Piezo1*^*WT*^ and *Piezo1*^*–/–*^ primary chondrocytes for experimentation. Finally, while our study employed COIP and SPR to biochemically validate the β-catenin/Lars2 interaction, the precise structural determinants mediating this intermolecular binding remain undefined. Specifically, the particular domain(s) of β-catenin (ARM repeats vs. N/C-terminal regions) orchestrating complex formation with Lars2 require systematic investigation through truncation mutagenesis and crystallographic analyses. This knowledge gap needs and merits systematic resolution in our ongoing investigations. In conclusion, our study demonstrates that Yoda1-mediated Piezo1 activation enhances mitochondrial function and promotes endochondral ossification via the β-catenin/Lars2 axis, accelerating fracture healing (Fig. S5). We propose that the Piezo1/β-catenin/Lars2 signaling axis has the potential to be a novel therapeutic target for accelerating fracture healing.

## Materials and methods

### Experimental animals

All in vivo experiments were conducted on 12-week-old male mice. Wild-type C57BL/6J mice were obtained from the SiPeiFu Biotechnology Co. Ltd. (Beijing, China). *Col2a1*-CreERT2 mice were obtained from Cyagen Biosciences Inc. (Guangzhou, China). *Col2a1*-CreERT2; *R26*-tdTomato gene mice were obtained from the Shulaibao Biotechnology Co. Ltd. (Wuhan, China). The *Piezo1*^*f/f*^ mice were provided by Professor Weiguo Zou (Chinese Academy of Sciences, University of Chinese Academy of Sciences, Shanghai, China). To generate *Piezo1*^*Col2a1*^ mice, *Piezo1*^*f/f*^ mice were mated with *Col2a1*-CreERT2 mice, and filial-generation 1 (F1) littermates were mated with each other. The genotypes of *Piezo1*^*Col2a1*^ mice were determined by genotyping all filial-generation 2 (F2) offspring mice.^45^ To produce *Col2a1*-CreERT2; *Piezo1*^*f/f*^; *R26*-tdTomato mice, *Piezo1*^*f/f*^ mice were mated with *Col2a1*-CreERT2; *R26*-tdTomato mice and F1 littermates were mated with each other. The genotypes of *Col2a1*-CreERT2; *Piezo1f/f*; *R26*-tdTomato mice were genotyped in all F2 offspring. The primers used for each genotype are listed in Table S2. All animal experiments were approved by the Ethics Committee of the Hebei Medical University (IACUC-Hebmu-P2022017).

### Femoral fracture model

The surgical procedure for the middle femoral fracture model was as follows: After isoflurane gas anesthesia (3% for induction and 1% for maintenance), the right lower limb was shaved using an electric shaver. After disinfection with alcohol, an incision was made in the anterolateral side of the right femur. The muscles were separated by sharp dissection to expose the middle femur and the patella. The femur was then cut using a number 10 scalpel blade, and a 23G stainless steel intramedullary pin was inserted retrograde through the intercondylar notch into the medullary cavity, maintaining parallel alignment with the femoral mechanical axis. The tail of the needle was carefully trimmed to prevent the movement of the patellofemoral joint. The periosteum remained intact, and the soft tissue was well protected. The wound was closed after fracture stabilization. The mice were revived under anesthesia and returned to their housing cages for free movement. For the gene mice, tamoxifen (75 mg/kg) was dissolved in corn oil and administered subcutaneously for 5 consecutive days to induce the deletion of Piezo1 in chondrocytes. The first injection was performed on the 5th day after surgery, as chondrocytes in the callus first appeared on postoperative day 5.^14^ The mice were euthanised, the skin and muscles were dissected, and the entire femur was exposed. The hip and knee joints were detached, the intramedullary needle removed, and complete femoral samples collected.

### ScRNA-seq and analysis

For the ScRNA-seq experiments in this study, we used callus tissues from *Piezo1*^*Col2a1*^ (*n* = 3) and *Piezo1*^*f/f*^ (*n* = 3) mice 14 days post-surgery. Enzymatic digestion was used to dissociate all the callus samples into suspensions of individual cells.^46^ Next, complementary DNA (cDNA) amplification, chromium library construction, and library sequencing were performed using Lianchuan BioTechnology (Hangzhou, China). scRNA-seq data analysis was conducted using the Seurat R package (version 3.1.4) as previously described.^46^ The scRNA-seq results were grouped and visualized using uniform manifold approximation and projection dimensionality reduction technology. DEGs were identified by setting the adjusted *P*-value threshold at 0.05 to define the DEGs. All scRNA-seq data were uploaded to the National Center for Biotechnology Information (NCBI) Gene Expression Omnibus database (accession number: GSE266774).

### Micro-CT analysis

The removed fresh femoral specimens were scanned using a SkyScan 1176 micro-CT instrument (Micro-CT; 50 kV, 500 μA, and 9 μm/pixel). Micro-CT scan images were 3D reconstructed using the NRecon software (version 1.6, SkyScan; Microphotonics Inc., Allentown, PA, USA). Quantitative analysis of the fracture callus tissue images was performed using CTAn software (version 1.9, SkyScan). The parameters for micro-CT analysis were bone volume (BV), total volume (TV), bone volume fraction (BV/TV), and trabecular thickness (Tb. Th.).

### Staining of tissue sections

Femoral samples were decalcified for 21 days using an EDTA decalcifying solution (EDTA, Solarbio Science & Technology, Beijing, China). After decalcification, samples were dehydrated and embedded in paraffin. A series of 4 μm consecutive paraffin-embedded sections were cut from each sample for HE, SO/FG, IHC, and IF staining. For Lineage tracing staining, we dehydrated the samples using 30% sucrose solution and performed frozen sectioning.

### HE staining

HE staining was performed using a commercial kit (G1120; Solarbio, Beijing, China) following standardized histopathological protocols. Tissue sections underwent sequential processing: (1) Deparaffinization through two xylene immersions (5 min each); (2) Rehydration in graded ethanol series (100%, 95%, 85%, 75%; 5 min per concentration); (3) Nuclear staining with Mayer’s hematoxylin (2 min) followed by differentiated bluing in running tap water (5 min); (4) Cytoplasmic counterstaining with eosin Y (1 min); (5) Rapid dehydration through reverse ethanol gradients; (6) Final clearing in xylene and mounting with neutral gum. Stained sections were imaged under a microscope.

### SO/FG staining

A modified Safranin O/Fast Green cartilage staining kit (G1371; Solarbio, Beijing, China) was used. Paraffin sections were dewaxed and rehydrated in water. A freshly prepared Weigert dye solution was applied for 5 min and then washed with water. The acidic differentiation solution was used for 15 s, followed by washing with distilled water for 10 min. The sections were then stained with a Solid Green staining solution for 5 min. Subsequently, the sections were quickly washed with a weak acid solution for 15 s to remove the residual green solid and dried. The sections were stained with a safranin staining solution for 5 min. Finally, the sections were dehydrated in 95% ethanol for 2–3 s, in absolute ethanol for 2–3 s, and then in absolute ethanol for 1 min. Xylene was used for transparency, followed by neutral gum sealing, and the slides were observed under a microscope.

### IHC staining

Paraffin sections were routinely dewaxed, rehydrated, immersed in EDTA antigen retrieval solution in a 55 °C water bath for 10 h. An appropriate amount of endogenous peroxidase blocker was added dropwise and incubated for 10 min at room temperature. Goat serum was added, and the mixture was blocked for 1 h at room temperature. Diluted primary antibody was added and incubated at 4 °C overnight. The following day, enzyme-conjugated goat anti-rabbit/mouse IgG polymer added dropwise. The sections were incubated at 37 °C for 20 min. Appropriate amount of freshly prepared DAB solution was added and incubated for 5-8 min at room temperature. Finally, the samples were counterstained with haematoxylin for 1 min until the nuclei appeared blue, and the staining was immediately stopped. The sections were washed with tap water, dehydrated using an ethanol gradient, cleared with xylene, sealed with neutral gum, and observed under a microscope. The primary antibodies used were Piezo1 (1:100, 15939-1-AP, Proteintech, USA), Lars2 (1:200, 17097-1-AP, Proteintech, USA), β-catenin (1:200, 51067-2-AP, Proteintech, USA), RUNX2 (1:200, sc-390351, Santa Cruz Biotechnology, USA), and Osteopontin (1:200, 22952-1-AP, Proteintech, USA).

### IF staining

IF staining was performed according to standard experimental protocols.^47^ The sections were dewaxed and rehydrated for antigen retrieval. Membranes were then incubated with the primary antibody overnight at 4 °C. The next day, the sections were rewarmed, washed with TBST, and incubated with fluorescent secondary antibodies for 1 h at room temperature. After washing the samples with TBST, the slides were sealed with DAPI anti-fluorescence mounting medium and observed under a fluorescence microscope. The detection markers in the IF staining experiment were Lars2 (1:200, 17097-1-AP, Proteintech, USA), RUNX2 (1:200, sc-390351, Santa Cruz Biotechnology, USA), and Osteopontin (1:200, 22952-1-AP, Proteintech, USA).

### Lineage tracing staining

After restoring the frozen sections to room temperature, wash them three times with TBST. Goat serum was added and blocked for 1 h at room temperature. After removing the blocking solution, add the diluted primary antibody Collagen I (Col1) (1:100, Ab270993, abcam, USA) 4 °C to spend the night. The next day, restore the slices to to room temperature, wash them three times in TBST, drip an appropriate amount of Alexa Fluor 488 secondary antibody (1:200, A0423, Beyotime, China), and incubate at room temperature for 1 h. After washing the samples with TBST, the slides were sealed with DAPI anti-fluorescence mounting medium and observed under a fluorescence microscope.

### Cell culture and osteogenic differentiation

*Piezo1*^*WT*^ and *Piezo1*^*–/–*^ ATDC5 cells were obtained from Cyagen Biosciences. Osteogenic differentiation of *Piezo1*^*WT*^ and *Piezo1*^*–/–*^ ATDC5 cells was performed following the protocol reported by Hendrickx et al.^11^ After 7 days of chondrogenic differentiation, the chondrogenic induction medium of *Piezo1*^*WT*^ and *Piezo1*^*–/–*^ ATDC5 cells was replaced with osteoblast induction medium. After 7 days of osteogenic induction, ALP staining was performed using a BCIP/NBT alkaline phosphatase chromogen kit (Beyotime). After 21 days of osteogenic induction, calcium deposition was visualized using Alizarin Red staining (Solarbio Science & Technology). RNA and protein samples were extracted from the cells and collected after 14 days of osteogenic induction.

### qPCR experiments

Total RNA was extracted from ATDC5 cells using the RNeasy RNA extraction kit (Thermo Fisher Scientific, CA, USA). Reverse transcription was performed using GoScript™ Reverse Transcription Mix and Oligo dT primers (Promega Corporation, Madison, WI, USA). The mRNA expression levels were analyzed by RT-PCR using a One Step RT-qPCR Kit (Sangon Biotech, Shanghai, China). Primers used for qPCR are listed in Table S3. The 2^−ΔΔCt^ method was used for data analysis.

### WB experiments

Gel electrophoresis was performed using 20 µg protein samples per well at 150 V for 60 min. The PVDF membrane was operated at 400 mA for 30 min. The membranes were then blocked for 2 h with 5% nonfat milk powder. Immediately after blocking, the primary antibody was added and incubated overnight at 4 °C. After incubation, the primary antibody was removed, and the membranes were washed three times in TBST, followed by incubation with the secondary antibody. Finally, chromogenic experiments were performed using ECL chemiluminescence. The gray values of the bands were quantified using ImageJ software. The primary antibodies used were Piezo1 (1:1 000, 15939-1-AP, Proteintech, USA), Lars2 (1:1 000, 17097-1-AP, Proteintech, USA), RUNX2 (1:1 000, sc-390351, Santa Cruz Biotechnology, USA), Osteopontin (1:1 000, 22952-1-AP, Proteintech, USA), β-catenin (1:1 000, 51067-2-AP, Proteintech, USA), Collagen Type I (Col1) (1:1 000, 14695-1-AP, Proteintech, USA), ALP(1:1 000, PA5-106391, Thermo Fisher Scientific, USA), OCN (1:1 000, DF12303, Affinity Bioscience, USA), Mfn1 (1:1 000, 13798-1-AP, Proteintech, USA), Mfn2 (1:1 000, 12186-1-AP, Proteintech, USA), OPA1 (1:1 000, 27733-1-AP, Proteintech, USA), DRP1 (1:1 000, 12957-1-AP Proteintech, USA), β-Tubulin (1:1 000, ab7291, Abcam, USA), β-actin (1:1 000, 20536-1-AP, Proteintech, USA), and GAPDH (1:1 000, 10494-1-AP, Proteintech, USA). The secondary antibody used was (1:2 000, sc-2357, Santa Cruz Biotechnology, Santa Cruz, USA).

### Cell counting kit-8 (CCK-8) assay

To examine the proliferation capacity of ATDC5 cells following Piezo1 knockout, we employed the Super-Enhanced Cell Counting Kit-8 (C0048S, Beyotime) for the assay. Briefly, *Piezo1*^*WT*^ and *Piezo1*^*-/-*^ ATDC5 cells (2 × 10^3^ cells/well) were resuspended and seeded into 96-well plates. After incubation for 48 h, the supernatants were removed and replaced with 100 μL of fresh medium containing 10 μL of CCK-8 solution. After incubation for 0 h, 1 d, 3 d, 5 d, 7 d, and 9 d at 37 °C in the dark, the absorbance was measured at 450 nm using a microplate reader.

### ROS staining

ROS analysis was accomplished by staining with CM-H_2_DCFDA (S0035S, Beyotime). The final concentration of CM-H_2_DCFDA was 5 μmol/L. The ATDC5 cells were cultured as described above, then treated with CM-H_2_DCFDA and Hoechst 33342 for ~30 min. Subsequently, the fluorescence of CM-H_2_DCFDA was examined using flow cytometer (Sony ID7000). The data underwent processing with FlowJo version 10.8.1.

### Cell immunofluorescence staining

Cells cultured on slides were fixed with 4% PFA and blocked with 5% bovine serum albumin (BSA). The primary antibody was diluted in 5% BSA and incubated overnight at 4 °C. After rinsing, the cells were incubated with a fluorophore-conjugated secondary antibody for 1 h and counterstained with DAPI in the dark for 5 min. Images were acquired on a laser-scanning confocal microscope.

### COIP experiments

For immunoprecipitation, the lysates were precipitated with the indicated antibody or control IgG for 2 h. The immunoprecipitated complexes were collected and then washed four times with 1 mL of precooled IP lysate. Each wash was followed by centrifugation at 2 000 × *g* at 4 °C for 10 min, and the supernatant was carefully discarded. After the final washing step, as much of the supernatant as possible was discarded. Then, 80 μL of 1× reduced loading buffer was added, and the sample was boiled for 10 min in boiling water. The sample was centrifuged at 1 000 × *g* and 4 °C for 5 min, and the supernatant was aspirated. Finally, 10 μL of the supernatant was used for WB detection.

### Surface plasmon resonance (SPR)

The SPR experiments were performed on a biacore T200 system at 25 °C. The β-catenin (HY-P74381, MCE, USA) (50 μg/mL) was immobilized onto a CM5 sensor chip (BR100530, Cytiva) by amine coupling, and the flow cell was equilibrated with the reaction buffer at a flow rate of 10 µL/min (1.0 × phosphate buffer, pH 7.4). Then, Lars2 protein at different concentration gradients (0.3125 μmol/L, 0.625 μmol/L, 1.25 μmol/L, 2.5 μmol/L, 5 μmol/L, 10 μmol/L) (Jinan Boshang Biotechnology Co., LTD) were injected over the chip, and the responses were recorded as resonance units (RU).^48^

### Mitochondrial correlation experiments

#### TEM experiments

The cells were centrifuged, and 2.5% glutaraldehyde fixative was carefully added along the wall of the centrifuge tube. After collection, samples were refrigerated at 4 °C for 4 h. To prepare the samples, serially increasing concentrations of ethanol were used, and they were embedded in a transparent resin to ensure adequate support and stability. The samples were then ultra-thin sectioned (50–100 nm) using an ultra-micro microtome. High-resolution imaging was performed using a Hitachi (Tokyo, Japan) TEM system (Spirit 80.0 kV) to observe the mitochondrial microstructure and ultrastructural details of the samples.

#### JC-1 experiments

The assay was performed using an enhanced mitochondrial membrane potential detection kit (JC-1) (C2003S, Beyotime). The cells were cultured in confocal dishes. After induction, 1 mL of cell culture medium + 1 mL of JC-1 staining solution was added and mixed thoroughly. The cells were incubated at 37 °C for 20 min. The supernatant was aspirated, and the cells were washed twice with JC-1 staining buffer. Next, 1 mL Hoechst 33342 staining solution (C1026, Beyotime) was added, and the cells were incubated at 37 °C in a cell incubator for 10 min. The cells were washed twice with PBS, 2 mL of the cell culture medium was added, and the samples were observed under a fluorescence microscope.

#### Mito-Tracker experiments

Mito-Tracker Red CMXRos (C1035, Beyotime) and Mito-Tracker Green (C1048, Beyotime) were used for this experiment, with Mito-Tracker Green being costained with MitoSOX Red (S0061S, Beyotime). The cell culture was prepared using confocal culture dishes. When the cells reached a certain density, the cell culture medium was removed, and 1 mL of the prepared Mito-Tracker Red CMXRos working solution was added. The cells were incubated at 37 °C for 20 min. After incubation, cells were washed twice with PBS. Next, 1 mL Hoechst 33342 staining solution (C1026, Beyotime) was added, and the cells were incubated at 37 °C in a cell incubator for 10 min. The cells were washed twice with PBS and fresh cell culture medium preincubated at 37 °C was added. Finally, the cells were observed under a confocal laser microscope.

#### MitoSOX experiments

The assay was performed using MitoSOX Red (S0061S; Beyotime). The cell culture was prepared using confocal culture dishes. When the cells reached a certain density, the cell culture medium was removed, and 1 mL of the prepared MitoSOX Red working solution was added. The cells were incubated at 37 °C for 30 min. After incubation, cells were washed twice with PBS. Next, 1 mL Hoechst 33342 staining solution (C1026, Beyotime) was added, and the cells were incubated at 37 °C in a cell incubator for 10 min. The cells were washed twice with PBS and immediately observed under a confocal laser microscope.

#### ATP concentration experiments

This experiment was performed using an Enhanced ATP Assay Kit (S0027, Beyotime). The cells were lysed by adding 200 µL of lysis buffer per well in a 6-well plate. After lysis, the cells were centrifuged at 12 000 × *g* at 4 °C for 5 min, and the supernatants were collected for subsequent assays. The ATP detection reagent was diluted with an ATP detection reagent dilution solution at a ratio of 1:4. Then, 100 µL of ATP test solution was added to each test well or tube. The solution was left at room temperature for 5 min to allow the consumption of background ATP and to reduce the background signal. Subsequently, 20 µL of the sample or standard was added to the test well or tube and quickly mixed using a micropipette. Chemiluminescence was detected using a multifunctional microplate reader.

#### Seahorse XF cell mitochondrial stress test and glycolytic rate assay

In this experiment, the OCR and ECAR were measured using an Agilent Seahorse XFe24 analyzer to characterize cellular mitochondrial function. The day prior to the experiment, the Seahorse XFe24 instrument host was turned on to warm it. The cells were seeded into the cell culture microplates of the XFe 24 analyzer at a density of 20 000 cells per well. Simultaneously, the probe plate is hydrated. On the day of the experiment, the cell culture medium was replaced with a basal medium (Agilent, USA) supplemented with 1 mmol/L pyruvate, 2 mmol/L glutamine, and 10 mmol/L glucose as nutrient substrates. The cells were then incubated in a CO_2_-free incubator at 37 °C for 60 min. The OCR experimental kit contained the following regulators: Oligomycin, FCCP, Rotenone, and Antimycin A.^49^ The hydrated probe plate was removed, and 15 μmol/L Oligomycin (56 µL) was added to well A, 40 μmol/L FCCP (62 µL) to well B, and 5 μmol/L Rotenone/Antimycin A (69 µL) to well C. The ECAR experimental kit contained the following regulators: Rotenone plus Antimycin A (Rot/AA) and 2-deoxy-D-glucose (2-DG). The hydrated probe plate was removed, and 5 μmol/L Rot/AA (56 µL) was added to well A, 500 mmol/L 2-DG (62 µL) to well B. The instrument was calibrated for ~20 min, after which the probe plate was switched to XFe24 cell culture plates.

#### Drug use

We used shRNA for cell transfection and AAV for in vivo transfection. Lars2-shRNA and Lars2-AAV targeting chondrocytes were designed and constructed by Shanghai Jikai Gene Technology Co., Ltd. Three shLars2 interference sequences were designed (Table S1) and cloned into a U6-MCS-CAG-EGFP vector to construct the plasmid. Through WB analysis, the shLars2 plasmid with the most effective interference was selected for subsequent cell experiments. The gene sequence of this plasmid was inserted into the *Col2a1*p-EGFP-mir155(MCS)-SV40 PolyA vector element to construct shLars2-AAV for in vivo experiments.

In this study, cell plasmid DNA transfection was performed using Lipo6000™ transfection reagent (C0526, Beyotime). Cells were seeded at ~400 000 cells per well the day before transfection, allowing the cell density to reach ~70%–90% the following day. The medium was replaced with 2 mL of fresh culture medium without antibiotics. Two centrifuge tubes were prepared, one containing 125 µL of DMEM without antibiotics or serum. To this tube, 2.5 µg of plasmid DNA was added and mixed gently using a micropipette. The second tube contained 5 µL Lipo6000™ transfection reagent, which was mixed gently with a micropipette. After incubation at room temperature for 5 min, the plasmid DNA solution was gently added to the tube containing Lipo6000™ reagent, and the mixture was gently pipetted. The solution was left at room temperature for 5 min before being added to the cells in the six-well plates. Gene silencing effects were evaluated using WB analysis.

In this study, the shLars2-AAV sample concentration was 3.41e + 12 v.g/mL, and 5e + 10 v.g of shLars2-AAV was administered on the 5^th^ day post-fracture.^50^ We performed in situ injections into the mouse callus tissue using a microsyringe, administering 15 μL per mouse.^13^ The postoperative day 5 time point was chosen for injection because chondrocytes begin to regenerate at that stage, allowing shLars2-AAV to specifically target these cells.^27^ Yoda1 (HY-18723, MedChemExpress) was used as a Piezo1-selective agonist in this experiment.^13^ Yoda1 was dissolved in 50 mmol/L DMSO and used for cell experiments.^13^ For femoral fracture mice, 5 μmol/kg Yoda1 was administered intraperitoneally 5 days per week.^13^ In this experiment, XAV939 (HY-15147, MedChemExpress) was used as an inhibitor of the Wnt/β-catenin signaling pathway.^51,52^ XAV939 was dissolved in a solution of 10% DMSO + 90% (20% SBE-β-CD saline) to produce a suspension of 1.56 mg/mL (5.00 mmol/L). For the femoral fracture mice, 30 mg/kg XAV939 was injected intraperitoneally 5 days per week.

#### Data processing

All experiments in this study were independently repeated three or more times, and the data are expressed as mean ± standard deviation. Data analysis and comparison were performed using GraphPad Prism software (version 9.5.1). For statistical analysis, the t-test was used to compare data between two groups, and a one-way analysis of variance was used to analyze and compare data between multiple groups. Differences were considered statistically significant when the *P* value was less than 0.05.

## Supplementary information

Supplementary Figure S1

Supplementary Figure S2

Supplementary Figure S3

Supplementary Figure S4

Supplementary Figure S5

Supplementary Information

## Data Availability

The data that support the findings of this study are available from the responding author upon reasonable request.
