# Supplementary figures and images for: Mechanism of Piezo1 regulating chondrocyte mitochondrial function and promoting fracture healing through β-catenin/LARS2 signaling pathway
Source: Bone Res. 2025 Sep 24;13:79. doi: 10.1038/s41413-025-00459-4 (PMC12460606; doi:10.1038/s41413-025-00459-4)

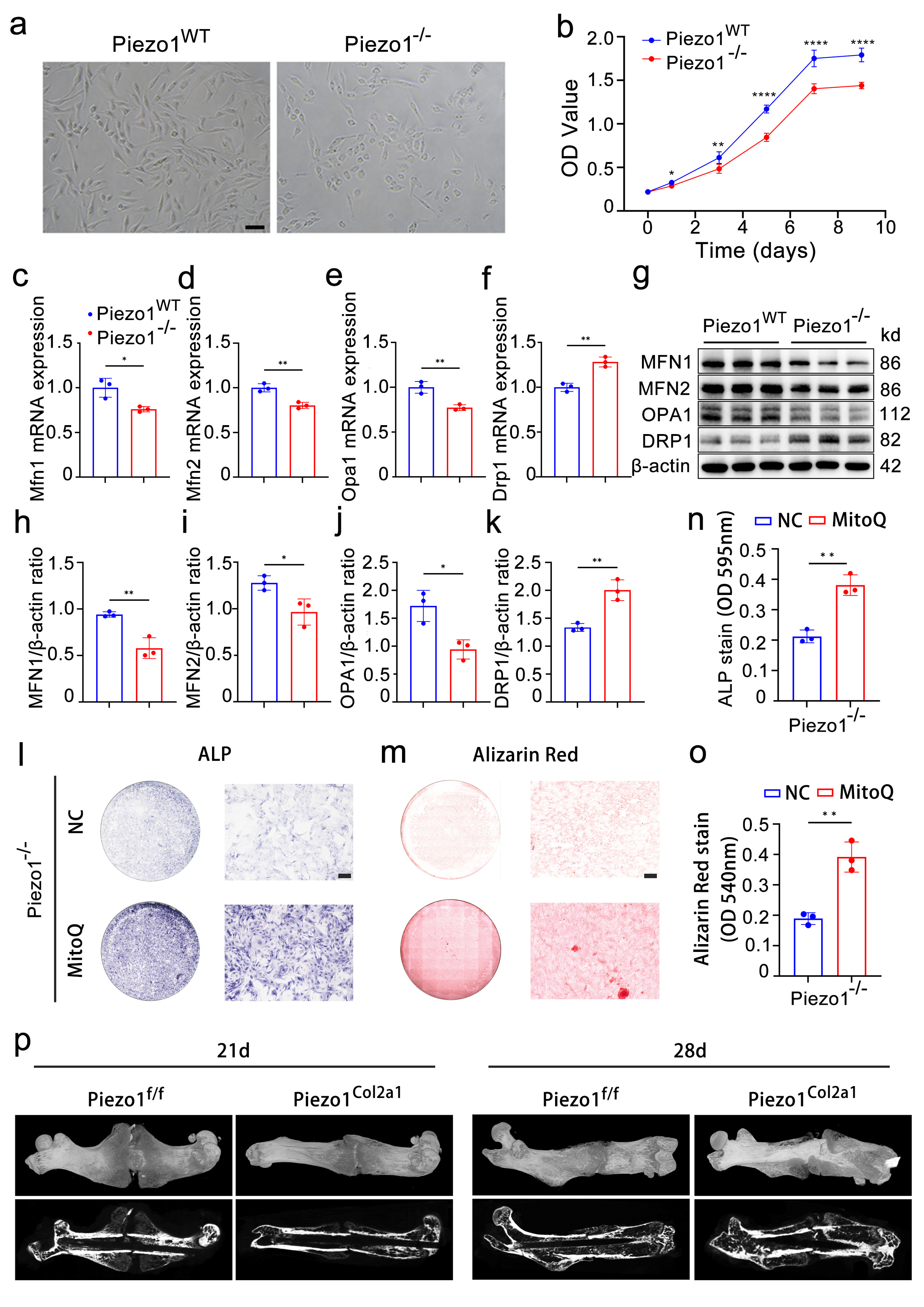

Supplement: Supplementary file 1 — Supplementary Figure S1 [file 41413_2025_459_MOESM1_ESM.jpg]

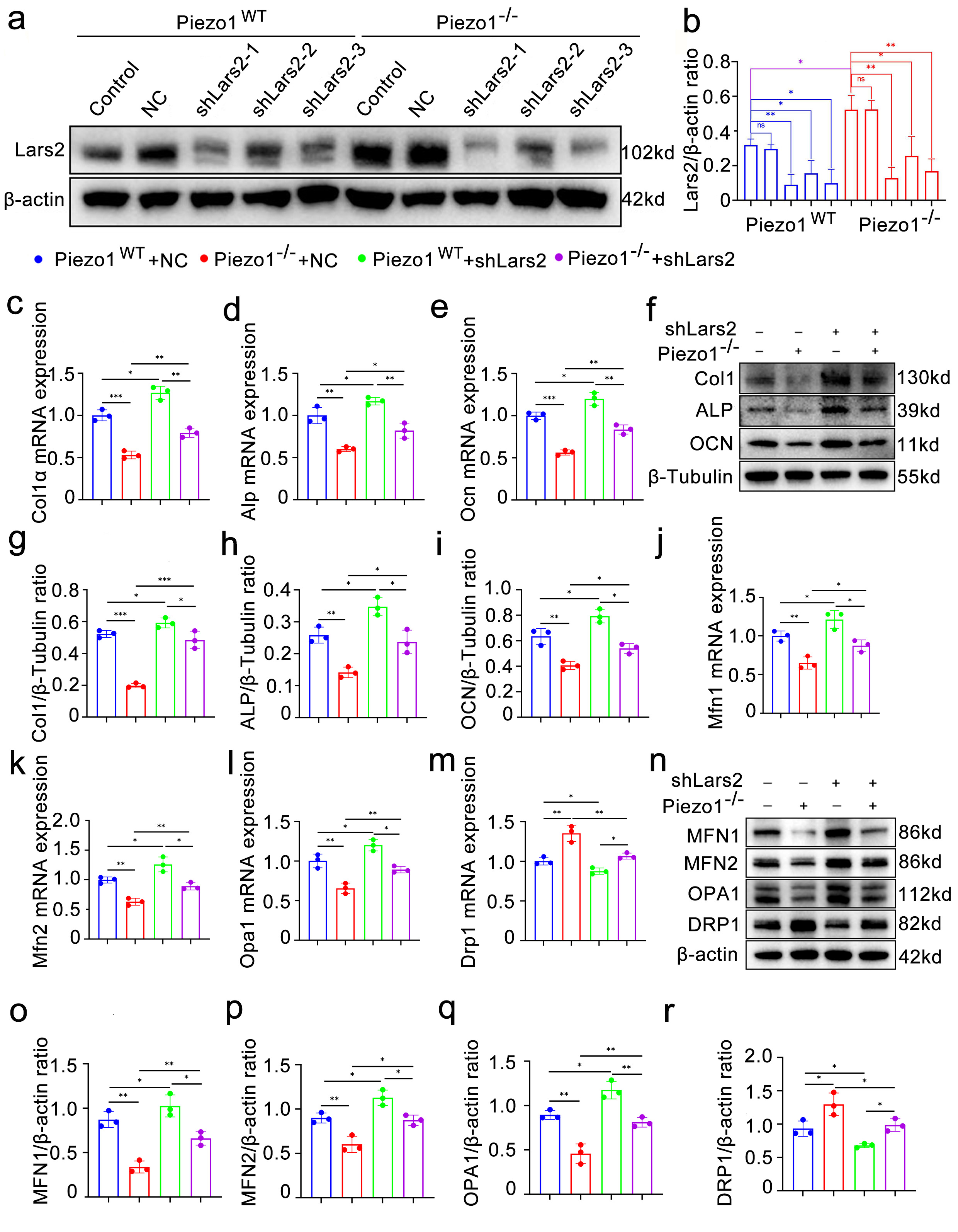

Supplement: Supplementary file 2 — Supplementary Figure S2 [file 41413_2025_459_MOESM2_ESM.jpg]

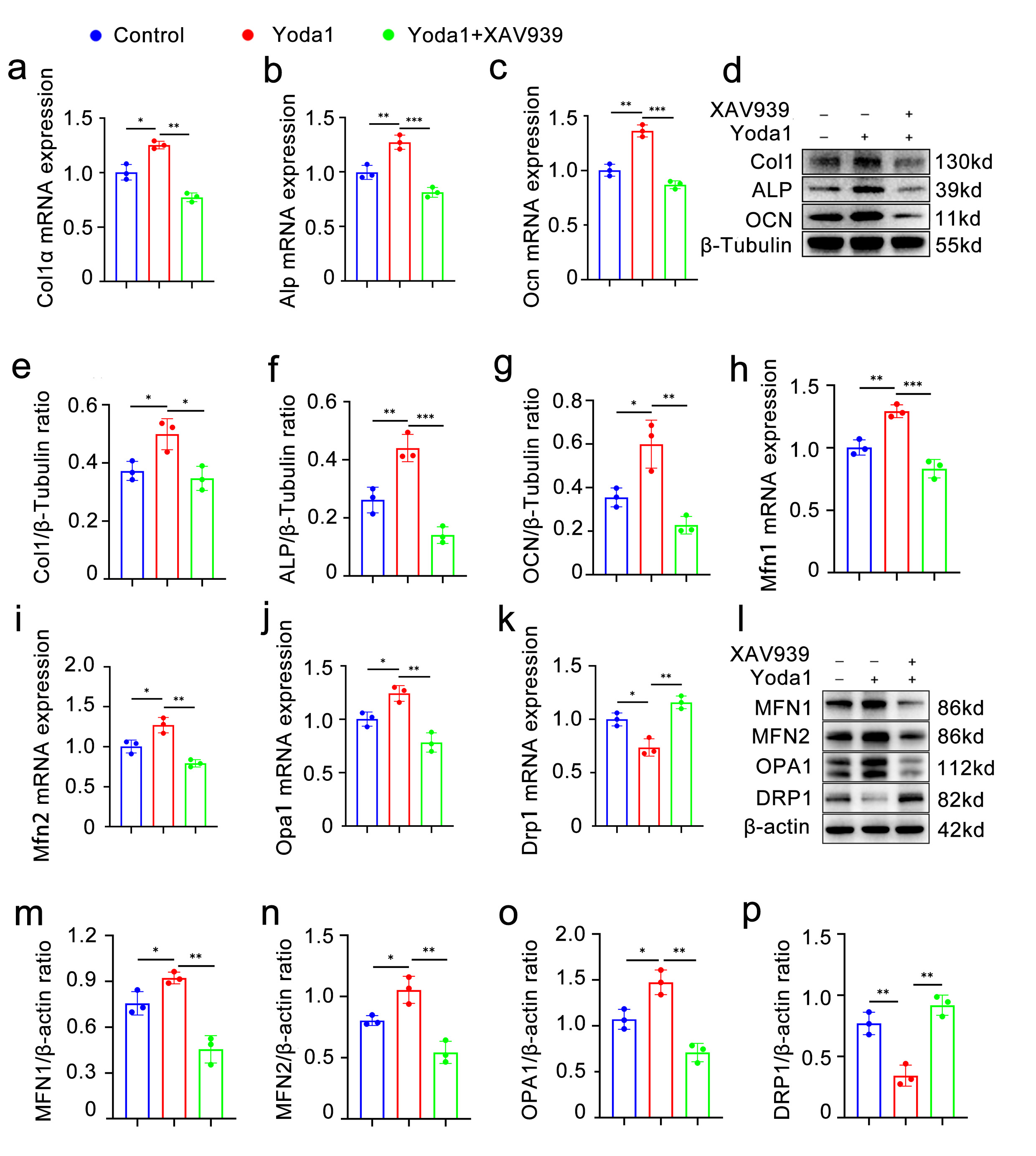

Supplement: Supplementary file 3 — Supplementary Figure S3 [file 41413_2025_459_MOESM3_ESM.jpg]

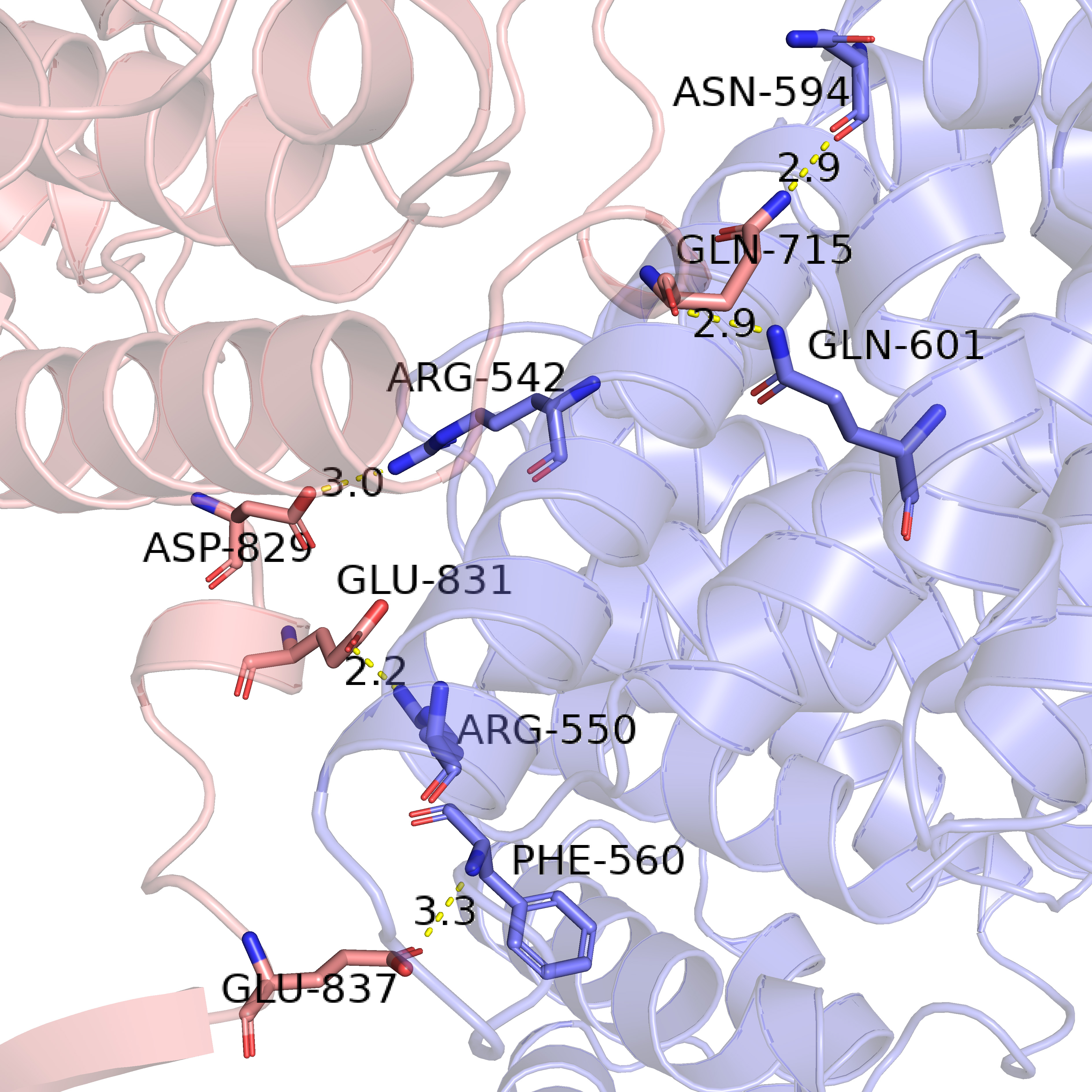

Supplement: Supplementary file 4 — Supplementary Figure S4 [file 41413_2025_459_MOESM4_ESM.jpg]

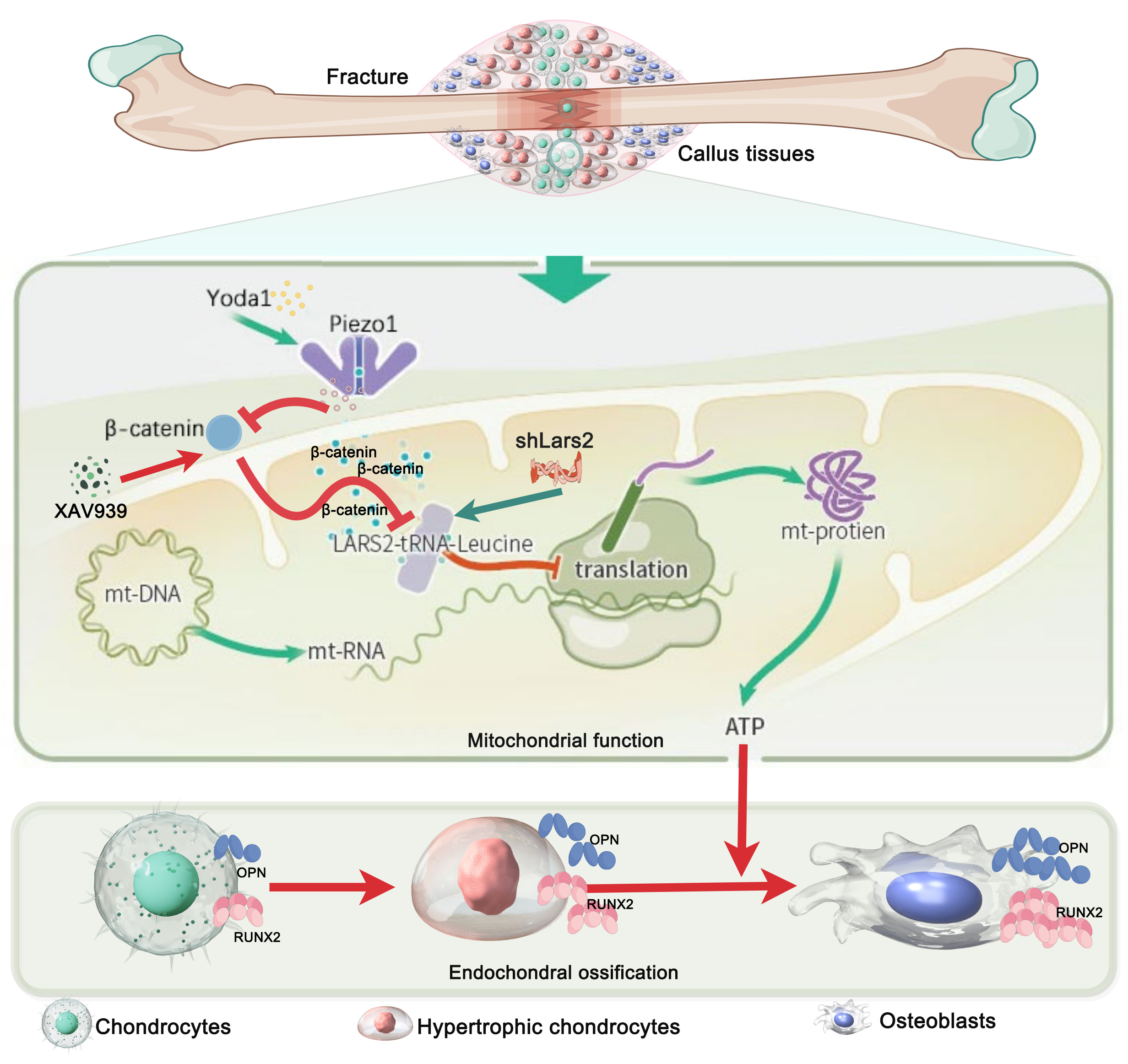

Supplement: Supplementary file 5 — Supplementary Figure S5 [file 41413_2025_459_MOESM5_ESM.jpg]
