# Supplementary material for: Mechanism of Piezo1 regulating chondrocyte mitochondrial function and promoting fracture healing through β-catenin/LARS2 signaling pathway
Source: Bone Res. 2025 Sep 24;13:79. doi: 10.1038/s41413-025-00459-4 (PMC12460606; doi:10.1038/s41413-025-00459-4)
Supplement: Supplementary file 6 — Supplementary Information [file 41413_2025_459_MOESM6_ESM.docx]

**Fig. S1** Phenotypic and functional alterations in chondrocytes following Piezo1 knockout. **a** Morphological observation of Piezo1*^WT^* and Piezo1^-/-^ ATDC5 cells were performed using optical microscope. **b** The proliferative capacities of Piezo1*^WT^* and Piezo1^-/-^ ATDC5 cells were quantitatively assessed using the CCK-8 assay. **c-f** qPCR was used to detect the expression of mitochondrial dynamics-related genes (Mfn1, Mfn2, Opa1, and Drp1) in Piezo1*^WT^* and Piezo1^-/-^ ATDC5 cells, n = 3. **g-k** WB was used to detect changes in the expression of mitochondrial dynamics-related markers (MFN1, MFN2, OPA1, and DRP1) in Piezo1*^WT^* and Piezo1^-/-^ ATDC5 cells, and statistical analysis, n = 3. **l, m** ALP and alizarin red staining were used to detect ALP levels after 7 days of osteogenic induction culture and the expression of calcium nodules after 21 days of induction culture, scale = 200 μm. **n, o** Statistical analysis of ALP and alizarin red stain were performed, n = 3. **p** Micro-CT observation of representative bone callus 3D reconstruction and coronal section images in each group 21 days and 28 days after femoral fracture. **P* < 0.05, ***P* < 0.01, and *****P* < 0.0001.

**Fig. S2** Inhibition of Lars2 ameliorated mitochondrial dysfunction in Piezo1^–/–^ ATDC5 cells and promoted their transdifferentiation into osteoblasts. **a, b** WB was used to determine the knockdown efficiency of shLars2 interference plasmids transfected with different sequences in each group, and statistical analysis was performed, n = 3. **c-e** qPCR was used to detect the expression of osteogenic genes (Col1α, Alp, and Ocn) in each group, n = 3. **f-i** WB was used to detect changes in the expression of osteogenic markers (Col1, ALP, and OCN) in each group, and statistical analysis, n = 3. **j-m** qPCR was used to detect the expression of mitochondrial dynamics-related genes (Mfn1, Mfn2, Opa1, and Drp1) in each group, n = 3. **n-r** WB was used to detect changes in the expression of mitochondrial dynamics-related markers (MFN1, MFN2, OPA1, and DRP1) in each group, and statistical analysis, n = 3. **P* < 0.05, ***P* < 0.01, and ****P* < 0.001.

**Fig. S3** Piezo1 can regulate Lars2 to affect mitochondrial function and promote endochondral ossification through β-catenin signaling. **a-c** qPCR was used to detect the expression of osteogenic genes (Col1α, Alp, and Ocn) in each group, n = 3. **d-g** WB was used to detect changes in the expression of osteogenic markers (Col1, ALP, and OCN) in each group, and statistical analysis, n = 3. **h-k** qPCR was used to detect the expression of mitochondrial dynamics-related genes (Mfn1, Mfn2, Opa1, and Drp1) in each group, n = 3. **l-p** WB was used to detect changes in the expression of mitochondrial dynamics-related markers (MFN1, MFN2, OPA1, and DRP1) in each group, and statistical analysis, n = 3. **P* < 0.05, ***P* < 0.01, and ****P* < 0.001.

**Fig. S4** Pymol 2.3.0 analysis of the docking model demonstrated that PHE-560, ARG-550, ARG-542, GLN-601, and ASN-594 of β-catenin formed hydrogen bonds with GLU-837, GLU-831, ASP-829, and GLN-715 of Lars2.

**Fig. S5** Graphic illustration of the findings of this study. Chondrocyte-specific Piezo1 ablation induces pathological upregulation of Lars2 expression, triggering mitochondrial bioenergetic failure that disrupts endochondral ossification dynamics. Conversely, genetic inhibition of Lars2 in chondrocytes restores mitochondrial oxidative phosphorylation capacity, thereby augmenting osteochondral differentiation potential. Mechanistically, pharmacological Piezo1 activation via Yoda1 rescues mitochondrial homeostasis through β-catenin-mediated transcriptional regulation of Lars2, which orchestrates fracture callus mineralization and accelerates osseous regeneration.

Table S1. Lars2 interferes with the gene sequence of the plasmid

| shRNA | Primer sequences |
| --- | --- |
| Lars2-RNAi-1(132836-1) | GCACAGGGAACCTTTCCATAA |
| Lars2-RNAi-2(132835-1) | GCCCAGCATTGCATCTTTAAC |
| Lars2-RNAi-3(132834-2) | GGTACGGAATCAAAGGCATGC |

Table S2. Genotyping Conditions and PCR Primers

| PrimerName | Primer sequence (5'-3') |
| --- | --- |
| Piezo1*^f/f^*-F | CCAGTGATTCCTCATGGAATGTGG |
| Piezo1*^f/f^*-R | CTTAAGCCCATCTCACAGCTGAAGG |
| *Col2a1*-CreERT2-F | GGCTCTACTTCATCGCATTCCTTG |
| *Col2a1*-CreERT2-R | CGCAAACAAGTCTCACAAAGGAG |
| Ai14-P1 | AAGGGAGCTGCAGTGGAGTA |
| Ai14-P2 | CCGAAAATCTGTGGGAAGTC |
| Ai14-P3 | GGCATTAAAGCAGCGTATCC |
| Ai14-P4 | CTGTTCCTGTACGGCATGG |

Table S3. Sequences of primers used for RT-PCR

| Gene | Forward primer (5′-3′) | Reverse primers (5′-3′) |
| --- | --- | --- |
| Piezo1 | GAATGTGATTGGGCAGCGTATGAAC | GAACAGCGTGAGGAACAGACAGTAG |
| Lars2 | ACCAGCTCAGAGGACACCAGAC | CGAGTCAGGGCTACAAAGGCATC |
| β-catenin | CCGTTCGCCTTCATTATGGACTAC | GGGCAAAGGGCAAGGTTTCG |
| OPN(SPP1) | TTCAATGGGCAGTTTTGAGC | ACTTACAAAACCGCCAAGC |
| RUNX2 | CCGCACGACAACCGCACCAT | CGCTCCGGCCCACAAATCTC |
| Col1a1 | AGGCGAACAAGGTGACAGAGG | GGAGAACCAGGAGAACCAGGAG |
| Alp | AACTGATGTGGAATACGAACTGGATG | CATAGTGGGAATGCTTGTGTCTGG |
| Ocn | AAGCAGGAGGGCAATAAGGT | TTTGTAGGCGGTCTTCAAGC |
| Mfn1 | GTGGGCTGGAAACTAATCTCTGTC | AACTGCTGCTTAAACGCTCTCTC |
| Mfn2 | GTGGTCGGAGGAGTGGTGTG | GTGCCAGGTCAGTCGCTCATAG |
| Opa1 | ATGACAGAACCCAAAGGAAAGGAAC | CCACTTGTGCCGCTTGATACTC |
| Drp1 | TGGCAACATCAGAAGCACTCAAG | TGGCATCAGTACCCGCATCC |
| GAPDH | GGGGAGCCAAAAGGGTCATCATCT | GAGGGGCCATCCACAGTCTTCT |
